# Spin pinning effect to reconstructed oxyhydroxide layer on ferromagnetic oxides for enhanced water oxidation

**DOI:** 10.1038/s41467-021-23896-1

**Published:** 2021-06-15

**Authors:** Tianze Wu, Xiao Ren, Yuanmiao Sun, Shengnan Sun, Guoyu Xian, Günther G. Scherer, Adrian C. Fisher, Daniel Mandler, Joel W. Ager, Alexis Grimaud, Junling Wang, Chengmin Shen, Haitao Yang, Jose Gracia, Hong-Jun Gao, Zhichuan J. Xu

**Affiliations:** 1 School of Materials Science and Engineering, Nanyang Technological University, Singapore, Singapore; 2 Beijing National Laboratory for Condensed Matter Physics and Institute of Physics, Chinese Academy of Science, Beijing, China; 3 Solar Fuels Laboratory and Energy Research Institute, Nanyang Technological University, Singapore, Singapore; 4 Talackerstrasse 9B, 5607 Hägglingen, Switzerland; 5 Department of Chemical Engineering, University of Cambridge, Cambridge, UK; 6 Institute of Chemistry, The Hebrew University of Jerusalem, Jerusalem, Israel; 7 Singapore-HUJ Alliance for Research and Enterprise (SHARE), Nanomaterials for Energy and Energy-Water Nexus (NEW), Campus for Research Excellence and Technological Enterprise (CREATE), Singapore, Singapore; 8 Department of Materials Science and Engineering, University of California at Berkeley, Berkeley, CA USA; 9 Berkeley Educational Alliance for Research in Singapore (BEARS), Ltd., Singapore, Singapore; 10 Chimie du Solide et de l’Energie, UMR 8260, Collège de France, Paris, Cedex France; 11 Réseau sur le Stockage Electrochimique de l’Energie (RS2E), CNRS FR 3459, Amiens, Cedex France; 12 MagnetoCat SL, General Polavieja 9 3I, Alicante, Spain; 13 Energy Research Institute @ Nanyang Technological University, 50 Nanyang Avenue, Singapore, Singapore

**Keywords:** Electrocatalysis, Electrocatalysis

## Abstract

Producing hydrogen by water electrolysis suffers from the kinetic barriers in the oxygen evolution reaction (OER) that limits the overall efficiency. With spin-dependent kinetics in OER, to manipulate the spin ordering of ferromagnetic OER catalysts (e.g., by magnetization) can reduce the kinetic barrier. However, most active OER catalysts are not ferromagnetic, which makes the spin manipulation challenging. In this work, we report a strategy with spin pinning effect to make the spins in paramagnetic oxyhydroxides more aligned for higher intrinsic OER activity. The spin pinning effect is established in oxide_FM_/oxyhydroxide interface which is realized by a controlled surface reconstruction of ferromagnetic oxides. Under spin pinning, simple magnetization further increases the spin alignment and thus the OER activity, which validates the spin effect in rate-limiting OER step. The spin polarization in OER highly relies on oxyl radicals (O∙) created by 1^st^ dehydrogenation to reduce the barrier for subsequent O-O coupling.

## Abstract (web summary)

Water oxidation to triplet oxygen requires a spin polarization process for faster kinetics. Here, the authors show an interface spin pinning effect between ferromagnetic oxides and reconstructed oxyhydroxide surface layer, where the spin ordering in paramagnetic oxyhydroxide catalyst layer can be tuned to improve the intrinsic activity.

## Introduction

In the process of realizing the hydrogen energy infrastructure, the application and optimization of technologies like water electrolyzers is critical. One of essential tasks for such application is the development of robust and low-cost catalysts for the oxygen evolution reaction (OER), which gives the major energy loss in water electrolysis^1,2^. Low-cost transition metal oxides (TMOs) are now intensively studied for efficient OER catalysis^1,3^. In a variety of studies, some Co-based perovskites and spinels have been found to undergo operando reconstruction to form active Co (oxy)hydroxides in alkaline condition during OER^4,5^. The di-μ-oxo bridged Co-Co sites in reconstructed (oxy)hydroxides induce the high OER activity after the deprotonation process, which generates the active oxygen ligand^6,7^. More effort has been made to explore reconstructable oxides and many excellent pre-catalysts have been developed^8,9^. According to the current study, the Co (oxy)hydroxides have been revealed as actual active species in the most reconstructable Co-based oxides in alkaline OER. Thus, leading the Co (oxy)hydroxides toward higher OER activity is now a critical step for developing high-performing OER pre-catalysts.

On the other hand, on the basis of current knowledge of OER, the OER kinetics is spin dependent^10–12^. In OER, the reactants including OH^−^ and H_2_O are singlet while the product oxygen has a triplet ground state with parallel spin alignment (↑0 = 0↑). The oxygen with triplet ground state was reported at a lower energy level of ~1 eV than its next excited state (the singlet oxygen)^13^. Thus, a spin polarization for producing triplet oxygen should be in a preferred path for good OER catalysts^14^. Considering the spin conservation for fast kinetics, the spin alignment in Co (oxy)hydroxides is critical for facilitating the spin-dependent reactions in OER^10,11^. Note that for spin-dependent catalysis, spin selection, spin-dependent electron mobility, and spin potentials in activation barriers could be optimized as quantum spin exchange interactions (QSEI) introduce a significant reduction of the electronic repulsions in the active d-orbitals of catalysts^15^. The maximum kinetic rates occur at catalytic interfaces with dominant ferromagnetic (FM) electronic delocalization, ${\Delta H}_{\uparrow \downarrow \to \uparrow \uparrow }^{{{\rm{act}}}.{{\rm{FM}}}}={\Delta H}_{\uparrow \downarrow \to \uparrow \uparrow }^{{{\rm{act}}}.{{\rm{Non}}}-{{\rm{magnetic}}}}-{\Delta H}_{\uparrow \downarrow \to \uparrow \uparrow }^{{{\rm{FM}}}.{{\rm{QSEI}}}}$, where ${\Delta H}_{\uparrow \downarrow \to \uparrow \uparrow }^{{{\rm{act}}}.{{\rm{FM}}}}$ is the activation enthalpy in a general spin-selective step and $-{\Delta H}_{\uparrow \downarrow \to \uparrow \uparrow }^{{{\rm{FM}}}.{{\rm{QSEI}}}}$ can reduce the barriers because of QSEI^10,12^.

The understanding of the QSEI in catalysis is associated with the Goodenough–Kanamori rules that explain the dominant magnetic ordering observed in magnetic metal oxides^16^. The dominant FM orderings of FM catalysts can be easily achieved as the magnetic moments in materials align along with the applied external magnetic field. A magnetic field-enhanced OER has been found recently in some FM oxides^14^, indicating the critical role of spin alignment in OER catalysis. It is also noted that some Co (oxy)hydroxides^17^, especially for those evolving under in situ OER condition^4^, have been reported among the best non-noble metal-based catalysts in alkaline media. For aligning the spins in Co (oxy)hydroxides, it should be noted that the Co (oxy)hydroxides typically do not show extended FM orderings^18,19^, thus the need for extremely high operando magnetic field, which would be rather difficult to apply in water electrolysis systems. Alternatively, for Co (oxy)hydroxide, it would be easier to control its spin alignment through a strong interaction in the interface between FM materials and materials without long-range FM, which is known as spin pinning^20,21^. It would come from the strong chemical bond in the interface of the two materials, which creates a strong magnetic anisotropy field. At the interface, the atoms could form a unit spin system through a long-range exchange interaction^22^. However, the spin pinning effect, toward enhanced OER catalysis, has not yet been demonstrated to our knowledge; and it is also challenging to realize the spin pinning in Co (oxy)hydroxide, especially difficult in reconstructable pre-catalysts.

Here we report a high-performing oxide_FM_/oxyhydroxide system where the spin pinning has been realized intrinsically in Co oxyhydroxide layer on FM Co_*x*_Fe_3−__*x*_O_4_ substrates (oxide_FM_/oxyhydroxide). Under spin pinning effect, the spins can be further aligned after a short-time magnetization under magnetic field, which further increases the OER activity. The spin pinning effect benefits from a stable oxide_FM_/oxyhydroxide interface and the long-range interaction is usually within 5 nm^20,22^. This calls for a stable oxide_FM_/oxyhydroxide configuration and an oxyhydroxide layer with limited thickness. We have achieved such stable oxide_FM_/oxyhydroxide configuration by a controllable reconstruction. The Co_*x*_Fe_3−__*x*_O_4_ oxides with controlled sulfurization degree can undergo surface reconstruction under OER condition to evolve Co oxyhydroxides layer in limited thickness of ~4 nm. At the interface, FM magnetic domains in Co_*x*_Fe_3−__*x*_O_4_ with highly aligned spins can result in a strong pinning of the spins in oxyhydroxide layer, for which the reconstructed oxyhydroxide exhibits higher intrinsic OER activity than directly prepared Co(Fe) oxyhydroxides by ~1 order of magnitude. After magnetization, the OER enhancement was notable only if the spin pinning had been established with FM substrates, and its effect can be turned OFF by demagnetization. This implies that the spin pinning effect is involved in the rate-determining step (RDS) of OER, and the RDS also believed to be spin-related. Under spin pinning, the 2p electrons in reactant oxygens with specific spin state (spin up or spin down) can transfer through catalysts during OER process, which creates oxygens with parallel spin alignment and thus promoting the production of triplet oxygen (↑0 = 0↑). Besides, it is also believed that the involvement of the oxyl radicals with more unpaired non-bonding p electrons in reconstructed oxyhydroxide is critical for the spin polarization in OER. As the oxyl radicals are created at the first dehydrogenation step, the spin polarization of oxygens can be facilitated accordingly under spin pinning, which reduces the barrier for the subsequent -O-O- coupling (RDS); otherwise to reach oxygen radicals with parallel spin alignment will cause additional barrier before O_2_ turnover. Overall, the design of oxide_FM_/oxyhydroxide system is based on a controllable surface reconstruction and introduces spin pinning effect to enhance OER.

## Result

### Controllable surface reconstruction

**Fig. 1:**
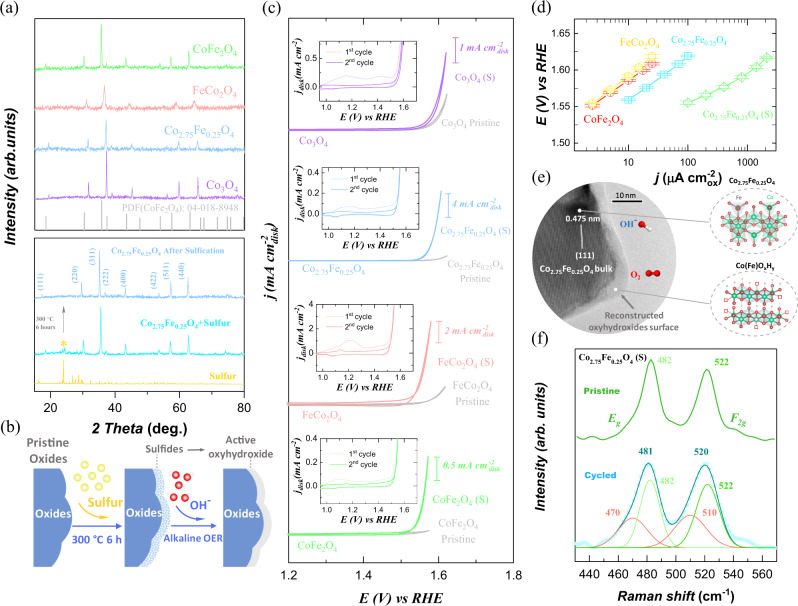
Controllable surface reconstruction on Co_3−__*x*_Fe_*x*_O_4_ spinel oxides for OER. **a** The powder X-ray diffraction (XRD) patterns of as-synthesized Co_3−__*x*_Fe_*x*_O_4_ (*x* = 0–2.0) (top) and Co_2.75_Fe_0.25_O_4_ as selected example before and after sulfurization (bottom). **b** The schematic diagram of a controllable reconstruction on oxides by controlling the sulfurization. The sulfurized products are denoted as Co_3−__*x*_Fe_*x*_O_4_ (s). **c** The cyclic voltammetry (CV) curves of Co_*x*_Fe_3−__*x*_O_4_ (*x* = 0–2.0) in O_2_-saturated 1 M KOH with a scan rate of 10 mV s^−1^. Inset is the first and second CVs of sulfurized oxides (noted as Co_3−__*x*_Fe_*x*_O_4_ (s)). **d** The Tafel plots of the OER specific activity of Co_2.75_Fe_0.25_O_4_ (s) versus pristine Co_3−__*x*_Fe_*x*_O_4_ oxides. The plots are given after oxide surface area normalization, capacitance correction, and iR correction. The error bars represent the standard deviation from three independent measurements. **e** The high-resolution transmission electron microscope (HRTEM) image of Co_2.75_Fe_0.25_O_4_ (s) after reconstruction (i.e., Co_2.75_Fe_0.25_O_4_/Co(Fe)O_*x*_H_*y*_). The HRTEM sample is from the electrode that was cycled without adding carbon. The bulk Co_2.75_Fe_0.25_O_4_ oxide is covered by an amorphous (oxy)hydroxide layer with thickness of ~4 nm. **f** The Raman spectra of Co_2.75_Fe_0.25_O_4_ (s) before and after operating under OER. The peaks at Raman shift of 482 and 522 cm^−1^ are assigned to *E*_g_ and *F*_2g_ mode in Co_2.75_Fe_0.25_O_4_ spinel. The broad peaks at Raman shift of 470 and 510 cm^−1^ are resulted by the bending and stretching of O-Co-O in amorphous Co(Fe)O_*x*_H_*y*_^34–36^.

The surface reconstruction of many catalysts under OER condition provides chances to enhance their OER performance as the surface oxyhydroxide species are evolved as the active sites for OER^4,23,24^. In the TMOs, the perovskite like Ba_0.5_Sr_0.5_Co_0.8_Fe_0.2_O_3−*δ*_ (BSCF) is well known for its high specific activity and reconstructability under alkaline OER^1,25,26^. Such high reconstructability originates from a high oxygen p state, strong metal–oxygen covalency, and lattice oxygen participation in OER^5^. The reconstructability can be even more notable in most metal chalcogenides, nitrides, and phosphides, which will undergo complete reconstruction to oxyhydroxide species under OER condition^27^. The alkaline reconstruction is a simple and effective way to generate highly active oxyhydroxide that could be applied in the spin pinning system. The pinning depth, which is affected by the long-range exchange, is usually within 5 nm and the pinning effect requires a stable oxide_FM_/oxyhydroxide interface^20,22^. Thus, it is critical to design controllable surface reconstruction with finally stable surface chemistry and limited oxyhydroxide layer. Our design starts with Co_3−__*x*_Fe_*x*_O_4_ spinels, which are stable during alkaline OER. The Co_3−__*x*_Fe_*x*_O_4_ (*x* = 0–2.0) were synthesized by a sol–gel method. The top panel in Fig. 1a gives all powder X-ray diffraction (XRD) patterns of Co_3−__*x*_Fe_*x*_O_4_. As observed, the diffraction peaks in their XRD patterns match well with standard cubic spinel (Fd-3m), indicating pure-phase spinel structure of Co_3−__*x*_Fe_*x*_O_4_. Then we performed low-degree sulfurization on Co_3−__*x*_Fe_*x*_O_4_ (the products denoted as Co_3−__*x*_Fe_*x*_O_4_ (s)). A small amount of sulfur was mixed evenly with Co_3−__*x*_Fe_*x*_O_4_ powder, followed by heat treatment under 300 °C for 6 h. As seen in the XRD patterns of Co_2.75_Fe_0.25_O_4_ + sulfur before and after heat treatment (bottom panel, Fig. 1a), the peaks attributed to sulfur disappear, which implies the completion of sulfurization. Please see details of sulfurization in the “Methods” section. The sulfurization degree is obtained according to the elemental ratio of Co_3−__*x*_Fe_*x*_O_4_ (s) by inductively coupled plasma optical emission spectrometry (ICP-OES) measurement and summarized in Supplementary Table 1. We assumed that the sulfurization would promote the reconstruction under OER. Indeed, as sulfur owns higher p state than oxygen and stronger M-S covalent bond than M-O^28,29^, it is more reactive for lattice sulfur than lattice oxygen in OER, which grants great structural flexibility for reconstruction. Considering this, it is reasonable that many metal sulfides were reported to undergo notable reconstruction into (oxy)hydroxides when serving as alkaline OER catalysts^30^. With the lattice sulfur at the surface of stable oxides, the reconstruction could be promoted at the surface and is under control by the sulfurization degree (Fig. 1b).

Our electrochemical results support this hypothesis. Figure 1c shows the cyclic voltammetric (CV; second cycle) curves of Co_*x*_Fe_3−__*x*_O_4_ (s) and pristine Co_*x*_Fe_3−__*x*_O_4_ in 1 M KOH (please see details for measurements in “Methods”). The inset panels exhibit the first and second CV cycles of Co_*x*_Fe_3−__*x*_O_4_ (s). It was found that the Co_*x*_Fe_3−__*x*_O_4_ (s) with low-degree sulfurization exhibit much larger pseudocapacitance in the first cycle than in the second cycle, indicating notable reconstruction in first cycle. Such reconstruction is, however, negligible on pristine Co_*x*_Fe_3−__*x*_O_4_ (Supplementary Fig. 1). Without sulfurization, the pristine Co_*x*_Fe_3−__*x*_O_4_ (e.g., CoFe_2_O_4_) could survive for at least 500 CVs in 1 M KOH with negligible surface reconstruction under the observation by high-resolution transmission electron microscopy (HRTEM; Supplementary Fig. 2a, b). The reconstruction of oxides after sulfurization is further investigated using the sulfurized Co_2.75_Fe_0.25_O_4_ with different sulfurization degrees (see details in Supplementary Note 1). Hence, we believe that a low-level sulfurization would be effective to control the surface reconstruction of oxides. After reconstruction, Co_*x*_Fe_3−__*x*_O_4_ (s) deliver much higher activity than pristine Co_*x*_Fe_3−__*x*_O_4_ oxides (Fig. 1c). By evolving the Co(Fe)O_*x*_H_*y*_ surface from the pristine materials of Co_2.75_Fe_0.25_O_4_ (s), FeCo_2_O_4_ (s), and CoFe_2_O_4_ (s), the activity increment after reconstruction is notable (>20 times at overpotential of 350 mV)). However, for pure Co_3_O_4_ without Fe involvement, the activity increase after reconstruction is about 2 times at overpotential of 350 mV. Thus, Fe component plays an important role in reconstructed oxyhydroxide, which is previously reported to promote the deprotonation process to generate active oxygen ligand, thus boosting the OER performance^9^. Overall, we have shown that the low-degree sulfurization is a simple but effective strategy for promoting the reconstruction of Co_*x*_Fe_3−__*x*_O_4_ oxides during alkaline OER. By adjusting the elemental ratio in pre-catalysts (like the ratio of Co and Fe in our case), the activity of reconstructed oxyhydroxide could be optimized. In Co_x_Fe_3−*x*_O_4_ (s), Co_2.75_Fe_0.25_O_4_ (s) exhibits highest specific OER activity after reconstruction (Supplementary Fig. 6). The activity Co_2.75_Fe_0.25_O_4_ (s) with and without mixing carbon is also examined (Supplementary Fig. 7a, b). Without carbon, the Co_2.75_Fe_0.25_O_4_ (s) after reconstruction still exhibited good performance, indicating its potential for practical applications. The specific OER activity of Co_2.75_Fe_0.25_O_4_ (s) in Tafel plots is compared to pristine Co_x_Fe_3−*x*_O_4_ oxides in Fig. 1d. The current density has been normalized to oxide surface area which is determined by Brunauer–Emmett–Teller (BET) measurements (Supplementary Fig. 8)^31^. In Tafel plots, the specific activity of Co_2.75_Fe_0.25_O_4_ (s) is superior to that of all pristine Co_x_Fe_3−*x*_O_4_. Note that we are able to get specific activity by normalizing current density to oxide surface area^32^ because we have controlled the reconstruction strictly on surface with limited depth. This is evidenced by investigating the reconstructed Co_2.75_Fe_0.25_O_4_ (s) under HRTEM. In the HRTEM image of Co_2.75_Fe_0.25_O_4_ (s) after 20 cycles (Fig. 1e), we observed an amorphous oxyhydroxide surface with uniform thickness of ~4 nm on Co_2.75_Fe_0.25_O_4_ bulk. The Raman spectra of Co_2.75_Fe_0.25_O_4_ (s) before and after cycling are also presented in Fig. 1f. In top curve, two peaks at Raman shift of 482 and 522 cm^−1^ are assigned to *E*_g_ and *F*_2g_ mode in pristine Co_2.75_Fe_0.25_O_4_ spinel^33^. After cycling (bottom curve), these two peaks are broadened and with lower Raman shift, which is resulted by additional features of bending and stretching of O-Co-O in amorphous Co(Fe)O_*x*_H_*y*_ (at 470 and 510 cm^−1^)^34–36^. The controllable reconstruction with limited oxyhydroxide layer still brings remarkable activity enhancement because the electrochemical reactions are sensitive to surface chemistry. In another word, the reconstructed oxyhydroxides layer with limited thickness are efficiently used for OER enhancement but not compromising the bulk stability. This also meets the need to reach a stable oxide_FM_/oxyhydroxide configuration for realizing spin pinning effect.

### Spin pinning effect toward OER enhancement

**Fig. 2:**
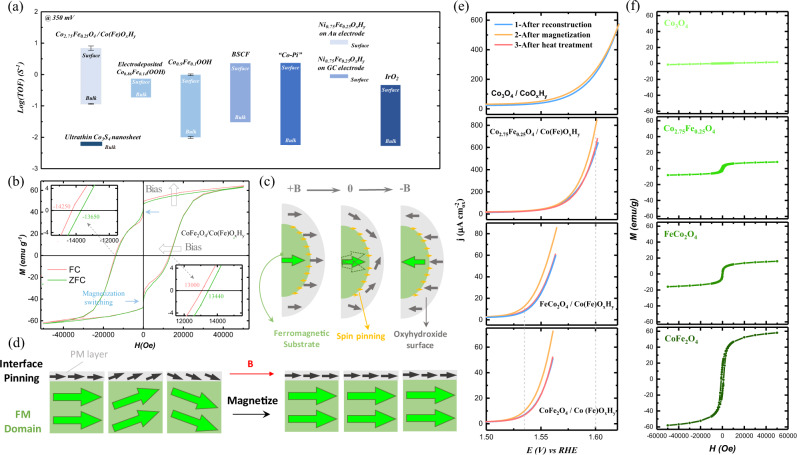
OER enhancement by spin pinning in Co_3−__*x*_Fe_*x*_O_4_/Co(Fe)O_*x*_H_*y*_ under magnetic field. **a** Turnover frequency (TOF) values of reconstructed Co_2.75_Fe_0.25_O_4_(s) (i.e., Co_2.75_Fe_0.25_O_4_/Co(Fe)O_*x*_H_*y*_), electrodeposited Co_0.86_Fe_0.14_(OOH)^17^, Co_0.9_Fe_0.1_OOH, Ba_0.5_Sr_0.5_Co_0.8_Fe_0.2_O_3−*δ*_ (BSCF) film^64,65^, electrodeposited cobalt hydroxide (Co-Pi)^65,66^, Ni_0.75_Fe_0.25_O_*x*_H_*y*_ NPs on Au electrode^53^, electrodeposited Ni_0.75_Fe_0.25_O_*x*_H_*y*_ on glassy carbon (GC) electrode^54^, IrO_2_ (in acid)^55^, and ultrathin Co_3_S_4_ nanosheet^67^. The TOF_bulk_ and TOF_surface_ of some catalysts present the lower and upper limits of the estimated TOF for fair comparison. The methods for TOF evaluation are given in the “Methods” section. The error bars represent the standard deviation from three independent measurements. **b** The magnetic hysteresis loops of CoFe_2_O_4_ (s) after 10 OER CVs (i.e., CoFe_2_O_4_/Co(Fe)O_*x*_H_*y*_) under both field-cooled (FC) mode and zero-field-cooled (ZFC) mode. The inset provides enlarged panels showing the loops that intersect with *y* = 0 axis. **c** The schematics of the exchange bias under switching magnetic field, which is closely associated with the spin pinning effect at the interface of ferromagnetic substrate/oxyhydroxide. **d** The schematic illustration of the spin pinning effect at the interface between ferromagnetic (FM) magnetic domains and the thin paramagnetic (PM) oxyhydroxide layer, and the spins in the PM oxyhydroxide layer can be aligned more under magnetization. **e** Linear sweep voltammetry (LSV) of Co_3−*x*_Fe_x_O_4_ (s) after reconstruction in 1 M KOH following the procedures: (1) after reconstruction (10 CV cycles in 1 M KOH, light blue), (2) after magnetization under 0.5 T for 15 min and the removal of the magnetic field (yellow), and (3) after the post-treatment at 120 °C for 1 min (pinkish). The gray dash lines denote the OER potential where the current density has been improved by 20% compared to that before magnetization. The error bars represent the standard deviation from three independent measurements given in Supplementary Fig. 17. **f** Magnetic hysteresis loops of Co_3−*x*_Fe_x_O_4_ oxides.

By controlling a low-level sulfurization of pre-catalysts, we have successfully realized a stable oxyhydroxide surface with limited thickness (~4 nm) on FM Co_3−*x*_Fe_x_O_4_ oxides (oxide_FM_/oxyhydroxide) under alkaline OER. To study the spin pinning effect in Co_3−*x*_Fe_x_O_4_/Co(Fe)O_*x*_H_*y*_, the intrinsic OER activity of the reconstructed Co(Fe)O_*x*_H_*y*_ was studied. The turnover frequency (TOF) of Co_2.75_Fe_0.25_O_4_/Co(Fe)O_*x*_H_*y*_ is shown in Fig. 2a, in comparison with the directly prepared Co(Fe) oxyhydroxides (one synthesized by us and one benchmark reported in literature; the Co/Fe ratio in oxyhydroxides is close to that in Co_2.75_Fe_0.25_O_4_). The bottom bound refers to the TOF by assuming that all metal cations in the catalyst are effective (denoted as bulk) and the upper one refers to the TOF by calculating only the active metal cations on the surface (denoted as surface). The details about the TOF evaluation are given in the “Methods” section. The details about the synthesis and characterizations of Co_0.9_Fe_0.1_OOH are provided in Supplementary Note 2. The as-synthesized Co_0.9_Fe_0.1_OOH shows comparable TOF_surface_ value with the benchmark Co_0.86_Fe_0.14_(OOH). Notably, the reconstructed Co(Fe)O_*x*_H_*y*_ exhibits TOF_surface_ of ~1 order of magnitude greater than pure Co(Fe) oxyhydroxide at 1.58 V (overpotential of 350 mV). The OER specific current densities of Co_2.75_Fe_0.25_O_4_/Co(Fe)O_*x*_H_*y*_ and Co_0.9_Fe_0.1_OOH (normalized to the BET surface area) are shown in Tafel plots (Supplementary Fig. 10). We find that Co_2.75_Fe_0.25_O_4_/Co(Fe)O_*x*_H_*y*_ consistently delivers higher specific activity (~1 order of magnitude) than Co_0.9_Fe_0.1_OOH at ~1.58 V. In addition, as compared with other well-known state-of-art OER catalysts, the TOF of Co_2.75_Fe_0.25_O_4_/Co(Fe)O_*x*_H_*y*_ on a per-surface-site basis is among the highest reported to date for alkaline OER catalysts.

To substantially evidence the spin pinning effect, we measured the magnetism of the reconstructed CoFe_2_O_4_ (s) (i.e., CoFe_2_O_4_/Co(Fe)O_*x*_H_*y*_) and the pristine CoFe_2_O_4_ under both field-cooled (FC) mode and zero-field-cooled (ZFC) mode. The magnetic hysteresis loops are shown in Fig. 2b and Supplementary Fig. 11. In the hysteresis loop of CoFe_2_O_4_/Co(Fe)O_*x*_H_*y*_, it exhibits large coercivity (*H*_C_) and a notable magnetization switching behavior at around zero field under both FC and ZFC modes^37,38^. However, the magnetization switching behavior is negligible for pristine CoFe_2_O_4_ while the high *H*_C_ is still notable. For CoFe_2_O_4_/Co(Fe)O_*x*_H_*y*_, the notable magnetization switching behavior at around zero field indicates that the CoFe_2_O_4_ substrate and the Co(Fe)O_*x*_H_*y*_ layer exhibit different magnetic properties, and the CoFe_2_O_4_ substrate serves as a strong ferromagnet leading to a high *H*_C_. Moreover, the loop of CoFe_2_O_4_/Co(Fe)O_*x*_H_*y*_, under FC mode, exhibits up and left shift compared to that under ZFC mode, while nearly no shift is observed in the hysteresis loop of pristine CoFe_2_O_4_. The up and left shift of CoFe_2_O_4_/Co(Fe)O_*x*_H_*y*_ under FC mode indicates an exchange bias effect that originates from uncompensated interfacial spins that are pinned in the oxyhydroxide layer and do not follow the external magnetic field (Fig. 2c)^39,40^. Such pinning effect is intrinsic. Under the electrochemical condition, the spin pinning can well persist. Seventy-two-hour chronopotentiometry test was performed on Co_2.75_Fe_0.25_O_4_/Co(Fe)O_*x*_H_*y*_ under OER condition, in which only limited activity drop is found (Supplementary Fig. 12a). The CVs before and after 72-h chronopotentiometry test show negligible difference (Supplementary Fig. 12b). CV cycling was also performed on Co_2.75_Fe_0.25_O_4_/Co(Fe)O_*x*_H_*y*_ and Co_2.75_Fe_0.25_O_4_ for 500 cycles in 1 M KOH (Supplementary Fig. 13a–d). The samples exhibit limited change since the second cycle during 500 CV cycles. Note that the first cycle involves the reconstruction from the pre-catalyst (sulfurized Co_2.75_Fe_0.25_O_4_) to the desired catalyst (Co_2.75_Fe_0.25_O_4_/Co(Fe)O_*x*_H_*y*_) and thus its CV profile is different from other subsequent cycles. In Supplementary Fig. 14, HRTEM was conducted on the Co_2.75_Fe_0.25_O_4_/Co(Fe)O_*x*_H_*y*_ after 500 CV cycles. The thickness of the oxyhydroxide layer still persisted its thickness after cycling. The thickness of the oxyhydroxide surface layer remained unchanged and thus the interface pinning effect should not be affected, which is consistent with the measured magnetic property. In Supplementary Fig. 15, the magnetic hysteresis loops of CoFe_2_O_4_/Co(Fe)O_*x*_H_*y*_ after 20 and 500 CV cycles show limited difference. The bias of hysteresis loop for the sample after 500 CV cycles still kept at ~1000 Oe. The evidence shows that the spin pinning persists in Co_3−*x*_Fe_x_O_4_/Co(Fe)O_*x*_H_*y*_ under the OER condition.

The intrinsic spin pinning effect led by the strong interface magnetic anisotropy originates from the existence of localized magnetic domains in the FM substrate. In each magnetic domain, the spins are highly aligned through exchange effects, which is well known as spontaneous magnetization for FM. The spin pinning usually happens when the magnetic domains are covered by compositions with disordered spins^21^, and the spin pinning effect could be found at the interface (Supplementary Fig. 16)^41,42^. Such pinning effect establishes under long-range exchange effect. Thus, when the oxyhydroxide layer is thin enough (at ~4–5 nm), the spins of the whole layer can be aligned by the underlying magnetic domains in the FM substrate. This is the case of our findings in the Co_3−*x*_Fe_x_O_4_/Co(Fe)O_*x*_H_*y*_, in which the Co_3−*x*_Fe_x_O_4_ is FM and the paramagnetic Co(Fe)O_*x*_H_*y*_ is a thin layer. The spins in reconstructed Co(Fe)O_*x*_H_*y*_ will be affected by a strong interface magnetic anisotropy and follow the spin ordering in the localized magnetic domains (Fig. 2d, left). However, not all magnetic domains in the FM materials are completely aligned in nature. That makes the spins in Co(Fe)O_*x*_H_*y*_ only aligned in part on the surface of localized magnetic domains before magnetization. After magnetization, magnetic domains can be aligned to establish a long-range FM ordering, which makes the spins in Co(Fe)O_*x*_H_*y*_ become more aligned along with the FM ordering (Fig. 2d, right). The spin ordering in the paramagnetic surface layer is thus further improved by magnetization.

It was then examined whether the enhanced spin alignment can further improve the OER. We performed OER linear sweep voltammetry (LSV) of Co_3−*x*_Fe_x_O_4_ (s) after following three procedures, respectively: (1) after the complete surface reconstruction; (2) after the magnetization under a magnetic field of 0.5 T for 15 min and then removal of the magnetic field; (3) after the post-treatment under 120 °C for 1 min. The results are shown in Fig. 2e. We also measured the magnetization curves of all substrate oxides (Co_3−*x*_Fe_x_O_4_) as shown in Fig. 2f. As seen in Fig. 2e, for reconstructed Co_3−*x*_Fe_x_O_4_ (s) (i.e., Co_3−*x*_Fe_x_O_4_/Co(Fe)O_*x*_H_*y*_), their OER performance can be further improved after magnetization with FM substrates like Co_2.75_Fe_0.25_O_4,_ FeCo_2_O_4_, and CoFe_2_O_4_. However, with Co_3_O_4_ substrate, which is paramagnetic at room temperature^43^, there is no activity enhancement after magnetization. It demonstrates that the magnetization can affect the OER efficiency of reconstructed oxyhydroxide surface layer only if the spin pinning was established by using a FM substrate. Besides, the activity enhancement for catalysts with Co_2.75_Fe_0.25_O_4,_ FeCo_2_O_4_, and CoFe_2_O_4_ substrates are also different. In Fig. 2a, the gray dash lines denote the OER potential where the current density has been improved by 20% compared to that before magnetization. For Co_2.75_Fe_0.25_O_4_, such improvement is observed at an OER potential of 1.60 V vs. reversible hydrogen electrode (RHE) while for FeCo_2_O_4_ and CoFe_2_O_4_ with higher magnetization than Co_2.75_Fe_0.25_O_4_, such improvement has already been notable at lower potential (1.53 V vs. RHE). Such activity enhancement strongly depends on the magnetization of Co_3−*x*_Fe_x_O_4_ substrate. To further confirm the observed OER enhancement, the Tafel plots using steady current by chronoamperometric test were conducted (Supplementary Fig. 18a, b). The result is consistent with those from LSV tests and the OER enhancement after magnetization is notable.

To check whether the enhancement of OER current density was simply resulted by the decrease of electrical resistivity of the oxide after magnetization, the CoFe_2_O_4_ with the highest FM among these oxides was tested for its magnetoresistance (Supplementary Fig. 19). Consistent with what has been previously reported for CoFe_2_O_4_^44^, it shows very limited change of the magnetoresistance after being magnetized under 0.5 T for 15 min. Further, a FM metal/oxyhydroxide configuration is constructed by cycling the Ni foil in 1 M KOH (Supplementary Fig. 20a, b). The activity enhancement of Ni foil can be observed after magnetization, and such activity enhancement by magnetization diminished after demagnetization (Supplementary Fig. 20c). As the Ni metal own high conductivity, the magneto-resistive effect can be excluded from the observed OER enhancement after the magnetization of metal/oxyhydroxide configuration. Moreover, it is also examined whether other electrochemical oxidation reactions in alkaline media such as methanol oxidation reaction (MOR) and formic acid oxidation reaction (FOR) can be enhanced by magnetizing the CoFe_2_O_4_/Co(Fe)O_*x*_H_*y*_ electrode (Supplementary Fig. 21a–d). If the OER activity enhancement by magnetization is resulted by magneto-resistive effect, the similar activity increase should be found in MOR and FOR as well. However, none of these reactions can be found with enhanced activity after magnetization. Other possible reasons for the activity enhancement by magnetization (e.g., magneto-hydrodynamic effect^45^) are also examined and excluded as discussed in Supplementary Note 3. Therefore, we believe that the magnetoresistance is not responsible for the observed OER enhancement. That OER shows unique activity enhancement by magnetization should be ascribed to unique OER steps (spin-related) from singlet reactant to the triplet product (↑0 = 0↑).

The magnetization improves the spin polarization in materials, which was previously reported to positively affect OER^14^. However, it is different in our case because the enhancement was observed after the magnetization, instead of in the presence of the external field. This is because the induced spin alignment by magnetization persisted for long even after the magnetic field was removed, which could be credited to the stable magnetization of FM substrate when magnetic field was removed (Supplementary Fig. 22)^46^. The OER enhancement of CoFe_2_O_4_/Co(Fe)O_*x*_H_*y*_ could preserve well in alkaline electrolyte for >2 h, as evidenced by the LSV result in Supplementary Fig. 23a. The induced magnetization completely relaxed finally and the OER activity of CoFe_2_O_4_/Co(Fe)O_*x*_H_*y*_ recovered to that before magnetization after overnight (~12 h) holding. But it should be noted that the catalysts still can be re-activated by magnetization again (Supplementary Fig. 23b). Besides, when the electrodes were heated at 120 °C for 1 min, the activity enhancement by magnetization soon diminished as the magnetic domains in Co_3−*x*_Fe_x_O_4_ become disordered by thermal disturbance, and the OER activity of Co_3−*x*_Fe_x_O_4_/Co(Fe)O_*x*_H_*y*_ returns to that before magnetization (Fig. 2e, pinkish lines). Overall, the turning ON/OFF effect of magnetization and its sensitivity to FM substrate demonstrate that the spin pinning effect indeed exists between the FM of Co_3−*x*_Fe_x_O_4_ substrate and the paramagnetic Co(Fe)O_*x*_H_*y*_ surface layer.

### Spin electrons in triplet oxygen production

**Fig. 3:**
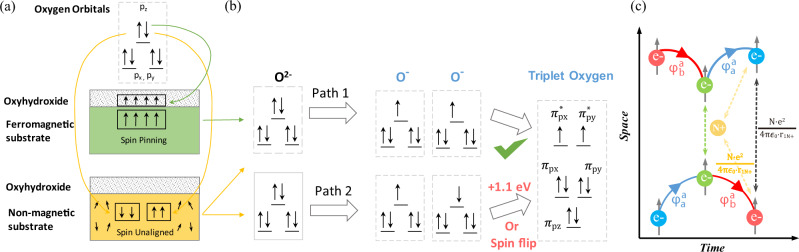
Spin pinning effect for triplet oxygen evolution on the oxyhydroxide. **a** The spin-electron transfer from singlet oxygen (OH^−^, H_2_O) to Co_3−*x*_Fe_x_O_4_/Co(Fe)O_*x*_H_*y*_ with and without spin pinning effect. **b** The triplet oxygen production by two oxygen radicals in parallel/opposite spin alignments. **c** The QSEI mechanism in a space–time Feynman diagrams. Two electrons with the same spin approach, in time from the left side, to avoid the increase of the Coulomb repulsions; the electrons exchange their orbitals (momentum) to effectively keep them apart. *φ* is the wavefunction of the orbitals (momentum) of spin electrons. The electronic repulsion between spin electrons is given as $\frac{{e}^{2}}{{4\pi {\epsilon }_{0}\cdot r}_{12}}$ and electron–nuclei Coulomb attraction is given as $\frac{{N\bullet e}^{2}}{{4\pi {\epsilon }_{0}\cdot r}_{1{N}^{+}}}$.^68^.

In OER, the reactants including OH^−^ and H_2_O are singlet while the product O_2_ has a triplet ground state; the singlet excited state of O_2_ is about 1 eV above the ground state^13^. The OER for producing triplet oxygen from singlet OH^−^ or H_2_O calls for a spin-selective electron transfer. In non-magnetic catalysts, the appropriate addition of spin-dependent potentials at the catalytic interphase would accelerate the kinetics and reduce the overpotentials through enhanced QSEI spin transfer^10^. In our case, the spin pinning effect acts to create intrinsic channels for the polarization of spin electrons within the catalysts. Schematics illustrating the spin pinning effect toward producing triplet oxygen are presented in Fig. 3a, b in a simple way. As shown in Fig. 3a, spin-up and spin-down electrons are paired in the p state of oxygen in singlet OH^−^ and H_2_O. The spins in substrate without FM are originally unaligned. Thus, both spin-up and spin-down electrons are allowed to transfer. However, when spins are aligned (spin-up) and pinned in oxyhydroxide by FM substrate, only spin-down electrons are allowed to pair with the spin-up electrons in oxyhydroxide. Such selective process under spin pinning would promote a spin polarization in OER process to facilitate the generation of triplet oxygen (↑0 = 0↑).

As shown in Fig. 3b, the OER process involve four electron-transfer steps, in which unpaired O 2p electrons can be created at first and third step. There are two possible paths (Path 1 and Path 2) to reach the Co-OO intermediate, depending on the spin electrons in oxygen p state. Path 1 will lead to a final production of triplet oxygen at lower energy of 1.1 eV than that of Path 2 for singlet oxygen, as revealed by our density functional theory (DFT) study (Supplementary Fig. 24). Alternatively, it will need spin flipping to align the spins of two oxygens before triplet O_2_ turnover, which would result in slow kinetics. Thus, for Path 2, the kinetics would be mostly blocked due to a high energy barrier required to give singlet oxygen^47^. For spin-unaligned substrate, the OER reactants take both Path 1 and Path 2 without spin selection, where the part of kinetics through Path 2 are blocked, and additional overpotential for spin polarization is required before the O_2_ turnover. However, for Co_3−*x*_Fe_x_O_4_/Co(Fe)O_*x*_H_*y*_ with spins aligned and pinned, the Path 1 for producing triplet oxygen is much preferred, which leads to lower kinetic barrier for O-O coupling. Thus, the OER enhancement of Co_3−*x*_Fe_x_O_4_/Co(Fe)O_*x*_H_*y*_ by spin pinning effect is closely associated with the facilitated spin polarization for triplet oxygen production.

Our analysis may also explain recent findings of OER enhancement by directly applying magnetic field on some FM oxides under operando condition^14^. It should be noted here the significant role of long-range FM orderings toward OER catalysis. In spin-aligned systems, the inter-atomic electronic repulsions between spin-oriented electrons will decrease to optimize the QSEI spin potentials. We show space–time Feynman diagrams in Fig. 3c to visualize the meaning of QSEI spin potentials. When two electrons with the same spin at some point in time are approaching each other (left side of Fig. 3c), to avoid the increase of the Coulomb repulsion, quantum mechanics allows only for electrons with the same spin to exchange their orbitals (momentum) to effectively keep them apart. As the right side of Fig. 3c shows, QSEI represent mechanisms that reduce the electronic repulsions but also imply a decrease of the electron–nuclei Coulomb attractions. Catalysts with dominant cooperative FM interactions, with an excess of degenerate empty valence orbitals, are stabilized via inter-atomic QSEI. The reduction of the electronic repulsions, dominant over Coulomb attractions, enhance the stabilization of electrons in the orbitals, also associated with extended spin mobility in dominant FM orderings. In relation with typical concepts in catalysis, QSEI make the stable Co_3−*x*_Fe_x_O_4_/Co(Fe)O_*x*_H_*y*_ catalytic interfaces to be more noble like, optimizing the spin-polarized kinetics^12^.

### pH-dependent OER enhancement by spin pinning

**Fig. 4:**
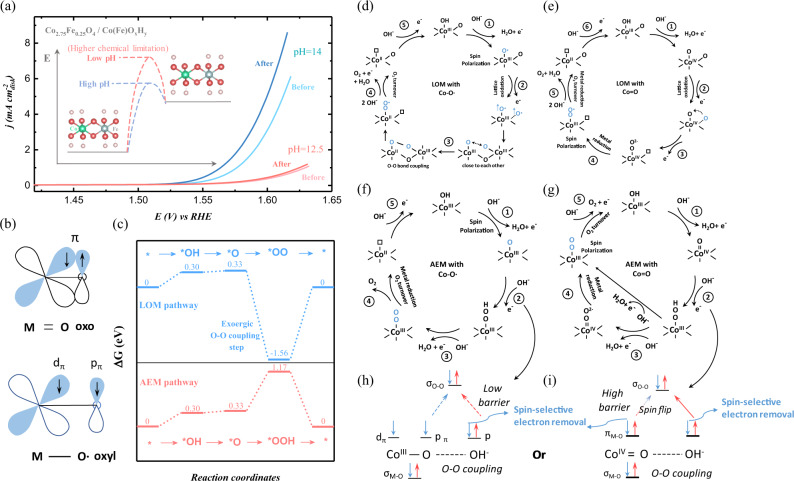
Active lattice oxygen participation for producing triplet oxygen. **a** The linear sweep voltammetry (LSV) of the reconstructed Co_2.75_Fe_0.25_O_4_ (s) (cycled in 1 M KOH for 10 cycles to evolved Co_2.75_Fe_0.25_O_4_/Co(Fe)O_*x*_H_*y*_) before and after magnetization in magnetic field of 0.5 T for 15 min under pH of 12.5 and 14. The inset is a schematic illustration of dehydrogenation under different pH for generating negatively charged oxygen on Co site. **b** The spin configurations of M = O oxo and M-O∙ oxyl species. **c** The free energy diagram of OER under AEM and LOM pathways at 1.23 V (vs. RHE) on β-Co(OOH) (001) slabs toward triplet oxygen production. **d**, **e** The LOM OER pathway for the spin polarization mechanism **d** with Co-O∙ oxyl radical and **e** with Co=O oxo species. The early spin polarization mechanism is closely associated with the creation of oxyl radicals. **f**, **g** The adsorbate evolution mechanism (AEM) OER pathway for the spin polarization mechanism **f** with Co-O∙ oxyl radical and **f** with Co=O oxo species. **h**, **i** The spin-related O-O coupling process under AEM **h** with Co-O∙ oxyl radical and **i** with Co=O oxo species.

We further investigated the OER enhancement by magnetization in alkaline media of different pH. Figure 4a shows the LSV of reconstructed Co_2.75_Fe_0.25_O_4_ (s) (i.e., Co_2.75_Fe_0.25_O_4_/Co(Fe)O_*x*_H_*y*_) before and after magnetization under pH of 12.5 and 14. It is clear that OER enhancement by magnetization is pH dependent. The influence of the resistance difference in the electrolytes with different pH has been excluded by iR-correction (Supplementary Fig. 25). The OER enhancement after magnetization is notable when performing OER in electrolyte of pH = 14, while such enhancement becomes much limited in electrolyte of pH = 12.5. As found in earlier study, the oxygen species on the surface of catalysts are highly dependent on the pH of electrolyte^48^. In high pH media, the ligand -OH is inclined to dehydrogenate and the left oxyl species are in great radical character. The radical oxygen ligands play a critical role in the O-O coupling process. It should be noted that the O-O coupling process with high activation energy can be a RDS in OER^49^. No remarkable OER enhancement after magnetization at low pH would be associated with the difficulty in dehydrogenation of −OH ligand to create −O• radical under low pH. Such pH-dependent OER enhancement by magnetization implies that the creation of oxyl species may be critical for the spin polarization of OER intermediates in OER. The spin configurations of metal oxyl and meal oxo species are shown in Fig. 4b. The M = O oxo has electrons paired in *π* orbitals, while the M-O∙ oxyl has two electrons in parallel spin alignment in d_*π*_ and p_*π*_ orbitals. Therefore, the creation of oxyl species leaves unpaired p electron on oxygen ligand. The spins of oxygen ligands highly depend on the spin ordering in metal sites. The FM spin ordering in metal sites under spin pinning can make the oxyl radicals become polarized, which is a prerequisite for spin polarization in OER.

As OER can be under adsorbate evolution mechanism (AEM) and lattice oxygen-mediated mechanism (LOM), we then discuss the role of oxyl radicals in spin polarization in OER under both pathways. The DFT was carried out to study the free energy diagram along with the AEM and LOM pathways (Fig. 4c). The details about DFT methods are given in Supplementary Information. Under AEM, the potential limiting step is the O-O coupling step. Whereas, under LOM, the O-O coupling process is down-hill in energy, indicating an exoergic process of O-O coupling^50^. The O-O coupling under LOM will thus be governed more by the kinetic barrier of this step.

For the OER steps with LOM, the spin polarization mechanisms are presented in the Fig. 4d, e. The critical step for the triplet oxygen production under LOM would be the formation of Co^III^-OO∙ intermediate, for which two oxygen radicals with parallel spin alignment are necessary (Supplementary Table 4). As the oxyl radical is induced in the first dehydrogenation step, the spin polarization of oxygen radical under LOM also can start at earlier OER step. Moreover, two oxygen radicals can be induced in the first and second electron transfer, in which one is from the ligand oxygen and another is from lattice oxygens. The two oxygen radicals can obtain parallel spin alignment under spin pinning before the formation of Co^III^-OO∙ intermediate. The two oxygen radicals next to each other with parallel spins allow spin exchange of these two oxygen radicals and two oxygens are close to each other, which finally leads to the formation of Co^III^-OO∙ intermediate. Without the oxyl radical after first dehydrogenation step, there is additional barrier as the spins of two oxygen need to be aligned first before the formation of Co^III^-OO∙ intermediate, which leads to slow kinetics for producing triplet oxygen. When a high activation energy of the O-O coupling step makes this step rate limiting, the spin polarization of oxyl radicals under spin pinning will increase the OER activity by reducing the kinetic barrier of O-O coupling.

Figure 4b, c present the spin polarization mechanisms with and without the generation of Co-O∙ oxyl radicals under AEM pathway. In the OER steps with AEM, the spin polarization of oxygen ligands under spin pinning also starts from the creation of the oxyl radical by dehydrogenation. The spin pinning effect will then benefit the subsequent O-O coupling step by facilitating spin-selective electron transfer. As shown in Fig. 4h, during the nucleophilic attack of OH^−^, the electron transfer with specific spin direction is facilitated, which leaves unpaired electrons in ∙OH radicals in opposite spin direction to that of electrons in Co-O∙ oxyl radicals. As the Co-OOH intermediates favor a singlet state with paired electrons (Supplementary Table 4), the O-O coupling can be facilitated because the electrons in ∙OH and Co-O∙ with antiparallel spin alignment pair with each other to generate σ_O-O_ bond. Alternatively, when spin-polarized electron is removed from Co-O∙ oxyl radicals and an empty orbital is created, a pair of electrons from OH^−^ can fill the empty orbital by nucleophilic attack to form σ_O-O_ bond (Supplementary Fig. 26a). Such O-O coupling with Co-O∙ oxyl radical can have a low barrier under spin pinning effect. However, without Co-O∙ oxyl radical, the *π* electrons in Co=O oxo species are paired. As shown in Fig. 4i, after the spin-selective electron removal from Co=O and the OH^−^, it will leave two electrons in parallel spin direction. To form σ_O-O_ bond, it will need to flip the spin direction of one electron, which leads to additional barrier for O-O coupling. Besides, as an alternative way toward σ_O-O_ bond formation (Supplementary Fig. 26b), the electron removal from *π*_M-O_ orbital to create an empty orbital is spin-unpolarized, which will still encounter a high barrier under spin pinning. Thus, considering the O-O coupling process as the rate-limiting step, the creation of oxyl radical with unpaired electron will be significant for improving the OER efficiency. Toward final O_2_ turnover, Co-OOH intermediate will need to remove electron from σ_O-H_ bond (dehydrogenation) and σ_M-O_ bond (M-OO dissociation). With the effect of spin-selective electron removal, two electrons with parallel spin direction will be left and leads to turnover of O_2_ in triplet state. Otherwise, if the dehydrogenation still gives Co-OO(2-) in singlet state (as indicated in Fig. 4g), it will need to overcome the additional barrier of spin flip to align the two oxygen radicals to generate Co-OO∙ radical.

In this study, the O-O coupling during OER is rate determining (energetically or kinetically), which could be facilitated by the spin polarization of oxyl radicals under spin pinning effect. As the generation of oxyl radicals is pH dependent, the magnetization effect on OER activity is also pH dependent. Similarly, as the spin pinning intrinsically exists, the pH dependence is also found in the intrinsic activity of Co_2.75_Fe_0.25_O_4_/Co(Fe)O_*x*_H_*y*_. But it should be also noted that such pH-dependent OER intrinsic activity could be also attributed to other non-concerted electron/transfer processes, such as refilling the surface oxygen vacancy with OH^−^.

To summarize, we have designed a controllable surface reconstruction on FM Co_3−*x*_Fe_x_O_4_ spinels by low-level sulfurization. After reconstruction under alkaline OER, the pre-catalysts Co_3−*x*_Fe_x_O_4_ (s) reached a stable Co_3−*x*_Fe_x_O_4_/Co(Fe)O_*x*_H_*y*_ configuration, where the oxyhydroxides layer is at limited depth of ~4 nm. Credited to the stable Co_3−*x*_Fe_x_O_4_/Co(Fe)O_*x*_H_*y*_ configuration with limited oxyhydroxides layer, the spin pinning effect has been introduced in oxyhydroxides, leading to higher intrinsic activity of reconstructed Co(Fe)O_*x*_H_*y*_ than directly prepared Co(Fe) oxyhydroxides by ~1 order of magnitude. With the spin pinning effect, simple magnetization can further enhance the OER performance of Co_3−*x*_Fe_x_O_4_/Co(Fe)O_*x*_H_*y*_ (*x* ≠ 0) as the pinned spins in Co(Fe)O_*x*_H_*y*_ layer become more aligned along with the long-range FM ordering of magnetic domains in Co_3−*x*_Fe_x_O_4_ substrate. Moreover, it is also believed that the generated oxyl radicals in reconstructed oxyhydroxide is critical for the spin polarization of ligand oxygen during OER. When it is generated in the first dehydrogenation step in high pH media, the ligand oxygens have more unpaired p electrons in non-bonding state and with more radical character. Under spin pinning, the spin polarization of the oxygen radicals can be facilitated, which reduces the barrier for subsequent O-O coupling. Overall, our design takes the advantage of a controllable surface reconstruction on FM oxides to realize the spinning pinning effect in Co oxyhydroxides. The usage of spin pinning effect is an effective way to stabilize extended FM orderings in active compositions, like Co oxyhydroxides in our case, and is a promising way of engineering QSEI in non-FM catalysts to improve catalysis. It is of great significance because in the family of excellent OER catalysts, many of them are hydroxides, oxyhydroxides, layer double hydroxides, and even some active perovskites that exhibit no long-range FM. The spin pinning effect provides them with a great potential for boosting spin-dependent kinetics to further enhance OER performance. Besides, the strategy toward controllable surface reconstruction of oxides would also contribute to designing high-performing pre-catalysts without compromising the bulk stability of reconstructed catalysts.

## Methods

### Co_3−*x*_Fe_x_O_4_ oxide synthesis

Co_3−*x*_Fe_x_O_4_ (*x* = 0, 0.25, 1, 2) spinel oxides were synthesized by a sol–gel method as described elsewhere^9^. Cobalt acetate (Co(OAc)_2_·4H_2_O) and Iron(III) nitrate nonahydrate (Fe(NO_3_)_3_·9H_2_O) were mixed in specific molar ratio according to the elemental ratio in products, then dissolved in the diluted nitric acid. Citric acid and urea were added into the solution, which was followed by stirring and heating up at 80–100 °С to form a viscous gel. The gel was dried and decomposed in air box at 170 °С for 12 h. Finally, the calcination was carried at 400–600 °С for 6 h to obtain Co_3−*x*_Fe_x_O_4_ (*x* = 0, 0.25, 1, 2) spinel oxides.

### Sulfurization of Co_3−*x*_Fe_x_O_4_ oxides

The as-prepared Co_3−*x*_Fe_x_O_4_ and sulfur powder were mixed in a mortar with a mass ratio of 10:1. The mixtures were sealed in a glassy tube under Ar atmosphere, which was followed by heating under 300 °C for 6 h. The sulfurization degree was decided by ICP-OES. The results are summarized in Supplementary Table 1.

### Material characterization

The XRD of Co_3−*x*_Fe_x_O_4_ were carried on Bruker D8 diffractometer at a scanning rate of 2° min^−1^, under Cu-K_α_ radiation (*λ* = 1.5418 Å). The BET measurement was conducted on ASAP Tristar II 3020 from single-point BET analysis. All samples were performed after 12 h outgassing at 170 °C. The BET surface area of Co_3−*x*_Fe_x_O_4_ oxides and their synthesis parameters are summarized in Supplementary Table 2. The HRTEM was carried JEOL JEM- 2100 plus microscope at 200 KV. The Fourier transform infrared spectroscopy–Raman spectroscopy was carried with a confocal Raman microscope (Labram HR EV0), equipped with a diode laser emitting at 532 nm at a nominal power of 12 mW. Laser power was limited at nominal 10% to avoid damaging samples. Spectra were recorded with the accumulation time of 60 s. For obtaining the Raman spectra of cycled catalysts, the catalysts were coated on carbon papers by drop casting for CV cycling in 1 M KOH (10 cycles), then dried under N_2_ gas before measurements.

### Electrochemical characterization

The working electrodes were fabricated by drop casting on glassy carbon electrodes. The powder samples were mixed with acetylene black at a mass ratio of 5:1, then were dispersed in isopropanol/water (v/v = 1:4) solvent followed by the addition of Na^+^-exchanged Nafion as the binder. The mixtures were ultrasonicated for 20 min to reach homogeneous ink. Before drop casting, the glassy carbon electrodes were polished to a mirror finish with α-Al_2_O_3_ (50 nm) and washed by IPA and water to completely clean up. Finally, the as-prepared ink (10 μl) was dropped onto glassy carbon electrodes (0.196 cm^2^) to reach a loading mass of 255 μg_ox_ cm^−2^ and the electrodes were dried overnight at room temperature_._ The electrochemical tests were conducted in a three-electrode system using Co_3−*x*_Fe_x_O_4_ electrode as working electrode, platinum plate (1 × 2 cm^2^) as the counter electrode, and Hg/HgO (1 M KOH, aqueous, MMO) as the reference. The CV and LSV are performed at a scan rate of 10 mV s^−1^ in O_2_-saturated 1.0 M KOH by using Bio-logic SP 150 potentiostat. All potentials measured are converted to RHE scale and under iR correction. The RHE reference calibration experiment was conducted by performing CV measurements in the voltage range of hydrogen electrocatalysis (HER/HOR) in H_2_-saturated 1 M KOH using Pt as the working electrode (Supplementary Fig. 28)^51^. The *E*_offset_ was obtained as the conversion factor: *E* (vs. RHE) = E (vs. Hg/HgO) − *E*_offset_ = *E* (vs. Hg/HgO) + 0.932 (V). The TOF was obtained according to the equation:

$${\rm{TOF}}=\frac{j\times A}{4{Fn}}$$

where *j* is the current density (A cm_disk_^−2^) delivered at an overpotential of 350 mV, *A* is the disk area of glassy carbon electrode (0.196 cm^−2^), *F* is the Faraday constant (96,485 C mol^−1^), and *n* is the number of active sites (mole). *n* is estimated by either assuming all active metal atoms in the active species are effective (denoted as “bulk”) or calculating only the active metal atoms on the surface (denoted as “surface”). The TOF_bulk_ and TOF_surface_ of some catalysts present the low and upper limit of the estimated TOF for fair comparison. The *n*_surface_ is obtained by an average integral area of the Co^2+^/Co^3+^ anodic and cathodic peaks (Supplementary Fig. 29). The *n*_bulk_ of the reconstructed Co(Fe)O_*x*_H_*y*_ is estimated by assuming all Co in the sulfurized Co_2.75_Fe_0.25_O_4_ surface have reconstructed into active oxyhydroxides, where the sulfurized degree of 2.06% is given by the ICP measurement. The *n*_surface_ is estimated by the integral area of the Co^2+^/Co^3+^ redox peaks (*A*_redox_) to obtain the population of Co that are electrochemically active on the surface (Supplementary Fig. 29). The *j* and *A*_redox_ of Co(Fe)O_*x*_H_*y*_ are obtained from the second cycle in CVs, followed by the correction of subtracting the background signals from Co_2.75_Fe_0.25_O_4_ substrate. The *n*_bulk_ of Co_0.86_Fe_0.14_(OOH) is reported to be estimated according to the measurement of the catalyst mass and composition by in situ QCM and ex situ XPS^17^. The *n*_surface_ is calculated by *n*_bulk_/*x*%, where *x*% is the fraction of Co in deposited Co_0.86_Fe_0.14_(OOH) that is electrochemically active (*x*% = 27%, second cycle)^17^. The *n*_bulk_ of BSCF film is reported to be calculated according to the unit cell volume, film thickness, and electrode area. The *n*_surface_ is reported to be determined by calculating the number of atoms on an assumed (100) surface according to the refined lattice parameters and the surface area of oxides that is decided by BET measurement. The *n*_surface_ of “Co-Pi” is reported to be obtained by the surface density of “Co-Pi” from extended X-ray absorption fine structure characterization^52^. The *n*_surface_ of Ni_0.75_Fe_0.25_O_*x*_H_*y*_ on both Au and GC electrode are reported to be estimated by the integral of the Ni^2+^ to Ni^3+/4+^ redox couple in CVs^53^. The reported *n*_bulk_ for Ni_0.75_Fe_0.25_O_*x*_H_*y*_ nanoparticles and electrodeposited Ni_0.75_Fe_0.25_O_*x*_H_*y*_ are very close to their *n*_surface_^53,54^. The *n*_surface_ of IrO_2_ is reported to be estimated according to refined lattice parameters with consideration of a (110) surface and the oxide surface area that is determined by BET measurement^55^. The *n*_bulk_ of BSCF, “Co-Pi”, IrO_2_, and Co_3_S_4_ are estimated by all active metal atoms in the catalysts loaded on the electrodes.

### Superconducting quantum design (SQUID) measurement

Direct current magnetization measurements were performed on a SQUID magnetometer (MPMS-XL). The Co_x_Fe_3−*x*_O_4_ powders were accurately weighted before measurements. The SQUID measurements of the magnetization of Co_x_Fe_3−*x*_O_4_ powders as a function of magnetic field were carried out at 300 K in fields between −5 and +5 T. For measuring the exchange bias of CoFe_2_O_4_/Co(Fe)O_*x*_H_*y*_, the CoFe_2_O_4_(s) samples were coated on carbon film by drop casting and following the same procedure as that in electrochemical measurements. After cycling, the samples were dried under N_2_ ambience before SQUID measurements. The SQUID measurement was then carried under FC (5 T) mode and ZFC mode and at a temperature of 2 K.

### Computational method

All the DFT calculations were performed by Vienna Ab-initio Simulation Package^56,57^, employing the Projected Augmented Wave^58^ method. The revised Perdew–Burke–Ernzerhof functional was used to describe the exchange and correlation effects^59–61^. For all the geometry optimizations, the cutoff energy was set to be 450 eV. A 4 × 2 × 1 Monkhorst–Pack grids^62^ was used to carry out the surface calculations on the layered oxyhydroxides. At least 20 Å vacuum layer was applied in *z*-direction of the slab models, preventing the vertical interactions between slabs.

In alkaline conditions, OER could occur in the following four elementary steps:

OH^−^ + * → *OH + e^−^

*OH + OH^−^ → *O + H_2_O + e^−^

*O + OH^−^ → *OOH + e^−^

*OOH +OH^−^ → * + O_2_ + H_2_O + e^−^

where * denotes the active sites on the catalyst surface. Based on the above mechanism, the free energy of three intermediate states, *OH, *O, and *OOH, are important to determine the OER activity of a given material. The computational hydrogen electrode model^63^ was used to calculate the free energies of OER. The free energy of the adsorbed species is defined as

$$\triangle {G}_{{{\rm{ads}}}}=\triangle {E}_{{{\rm{ads}}}}+\triangle {E}_{{{\rm{ZPE}}}}-T\triangle {S}_{{{\rm{ads}}}}$$

where ∆*E*_ads_ is the electronic adsorption energy, ∆*E*_ZPE_ is the zero point energy difference between the adsorbed and gaseous species, and *T*∆*S*_ads_ is the corresponding entropy difference between these two states. The electronic binding energy is referenced as ½ H_2_ for each H atom, and (H_2_O–H_2_) for each O atom, plus the energy of the clean slab. The corrections of zero point energy and entropy of the OER intermediates can be found in Supplementary Table 3. The slab models for calculating the free energy along with reaction coordinates are shown in Supplementary Fig. 27.

## Supplementary information

Supplementary Information in pdf

## Data Availability

The data that support the findings of this study are available from the corresponding author upon reasonable request.
