# Supplementary material for: Spin pinning effect to reconstructed oxyhydroxide layer on ferromagnetic oxides for enhanced water oxidation
Source: Nat Commun. 2021 Jun 15;12:3634. doi: 10.1038/s41467-021-23896-1 (PMC8206068; doi:10.1038/s41467-021-23896-1)
Supplement: Supplementary file 1 — Supplementary Information in pdf [file 41467_2021_23896_MOESM1_ESM.pdf]

Supplementary materials

Spin pinning effect to reconstructed oxyhydroxide layer on ferromagnetic oxides for enhanced water oxidation

Tianze Wu[1,2,3,Δ], Xiao Ren[1,Δ], Yuanmiao Sun[1], Shengnan Sun[1], Guoyu Xian[2], Günther G. Scherer[4], Adrian C. Fisher[5], Daniel Mandler[6,7], Joel W. Ager[8,9], Alexis Grimau[10,11], Junling Wang[1], Chengmin Shen[2], Haitao Yang[2], Jose Gracia[12], Hong-Jun Gao[2], Zhichuan J. Xu[1,3,13]**

[1]School of Materials Science and Engineering, Nanyang Technological University, 50 Nanyang Avenue, 639798, Singapore;

[2]Beijing National Laboratory for Condensed Matter Physics and Institute of Physics, Chinese Academy of Science, Beijing 100190, China;

[3]Solar Fuels Laboratory and Energy Research Institute, Nanyang Technological University, 50 Nanyang Avenue, 639798, Singapore;

[4]Talackerstrasse 9B, 5607 Hägglingen, Switzerland;

[5]Department of Chemical Engineering, University of Cambridge, Cambridge CB2 3RA, UK;

[6]Institute of Chemistry, The Hebrew University of Jerusalem, Jerusalem 9190401, Israel;

[7]Singapore-HUJ Alliance for Research and Enterprise (SHARE), Nanomaterials for Energy and Energy-Water Nexus (NEW), Campus for Research Excellence and Technological Enterprise (CREATE), 138602, Singapore;

[8]Department of Materials Science and Engineering, University of California at Berkeley, Berkeley, California 94720, USA;

[9]Berkeley Educational Alliance for Research in Singapore (BEARS), Ltd., 1 CREATE Way, 138602, Singapore

[10]Chimie du Solide et de l'Energie, UMR 8260, Collège de France, 75231 Paris Cedex 05, France;

[11]Réseau sur le Stockage Electrochimique de l'Energie (RS2E), CNRS FR 3459,33 rue Saint Leu, 80039, Amiens Cedex, France;

[12]MagnetoCat SL, General Polavieja 9 3I, 03012 Alicante, Spain;

[13]Energy Research Institute @ Nanyang Technological University, 50 Nanyang Avenue, Singapore 639798, Singapore.

[Δ]These authors contribute equally to this work.

*Corresponding authors: xuzc@ntu.edu.sg (Z. Xu), htyang@iphy.ac.cn (H. Yang)

Supplementary Note 1. The reconstruction can be controlled by the sulfurization degree of pristine oxides. The $Co_{2.75}Fe_{0.25}O_4$ was mixed with sulfur powder at mass ratios of 5:0.5, 5:1, and 5:3, followed by heat treatment for sulfurization. The sulfurization degree is estimated according to the ICP-OES measurement of sulfurized samples ($Co_{2.75}Fe_{0.25}O_4$ (s)) (Supplementary Table 1). The CVs (1st and 2nd cycle) of $Co_{2.75}Fe_{0.25}O_4$ (s) are given in Supplementary Fig. 3a-3f. The irreversible pseudocapacitive charge in the 1st cycle indicates the irreversible oxidation during the surface reconstruction of $Co_{2.75}Fe_{0.25}O_4$ (s). Such signal for reconstruction become more notable along with the increase of sulfurization degree. Accordingly, the reconstructed $Co_{2.75}Fe_{0.25}O_4$ / $Co(Fe)O_xH_y$ can deliver higher current density with higher-degree reconstruction. The reconstructed $Co_{2.75}Fe_{0.25}O_4$ (s) samples were observed under HRTEM (Supplementary Fig. 4a-4c). For all samples, an oxyhydroxide layer that is resulted by reconstruction can be found on the $Co_{2.75}Fe_{0.25}O_4$ substrate. In Supplementary Fig. 5, the thickness of the oxyhydroxide layer has been estimated and correlated linearly to the sulfurization degree of $Co_{2.75}Fe_{0.25}O_4$ (s).

Supplementary Note 2. As the activity of Co(Fe) oxyhydroxide is also sensitive to the Co/Fe ratio, we synthesized the $Co_{0.9}Fe_{0.1}OOH$ and referenced the benchmark $Co_{0.86}Fe_{0.14}OOH$[1] for the comparison with $Co_{2.75}Fe_{0.25}O_4$/Co(Fe)OOH (assuming that the Co/Fe ratio will not change remarkably after the surface reconstruction). The $Co_{0.9}Fe_{0.1}OOH$ was synthesized according to a reported hydrothermal method.[2] The HRTEM image and the FFT patterns of $Co_{0.9}Fe_{0.1}OOH$ are shown in Supplementary Fig. 9a and 9b. The steady CV of $Co_{0.9}Fe_{0.1}OOH$ at a scan rate of 10 mV s^{-1} in 1 M KOH is shown in Supplementary Fig. 9c. The surface area of $Co_{0.9}Fe_{0.1}OOH$ is indicated by BET measurement (Supplementary Fig.8e).

Supplementary Note 3. The measurement of $CoFe_2O_4$/$Co(Fe)O_xH_y$ was also performed in a two-electrode cell before and after magnetization. The OER performance of $CoFe_2O_4$/$Co(Fe)O_xH_y$ exhibits notable enhancement after magnetization (Supplementary Fig. 30a). This result excludes the possible influence of magnetization on the reference electrode. Moreover, magneto-hydrodynamic (MHD) effect under a magnetic field has been found to improve the mass transportation of ions near the electrode surface. The MHD effect originates from the Lorentz force that affects the direction of charged ions M^{x+} movement in electrolyte. The MHD effect decreases the thickness of the diffusion layer, which thus increases the limiting current in cathodic reactions such as metal electrodeposition. However, such an effect on ions may not be applicable to OER in aqueous solution. It has been well understood that OH^- in aqueous solution do not move physically, but by sequential proton hoping, known as Grotthuss mechanisms (Supplementary Fig. 30b)[3]. That means there is actually no physical movement of OH^- ions and thus the influence of Lorentz force on the OH- or H_3O+ is negligible. It can be excluded that the observed enhancement is from the MHD effect. In addition, it has been known that the MHD

effect can promote the gas bubble release and thus improve the HER and OER kinetics. However, that was under the high overpotential and high current region, in which the gas bubble release significantly affects the reaction kinetics.[4-5] It is not the case in the experiments presented in this work.

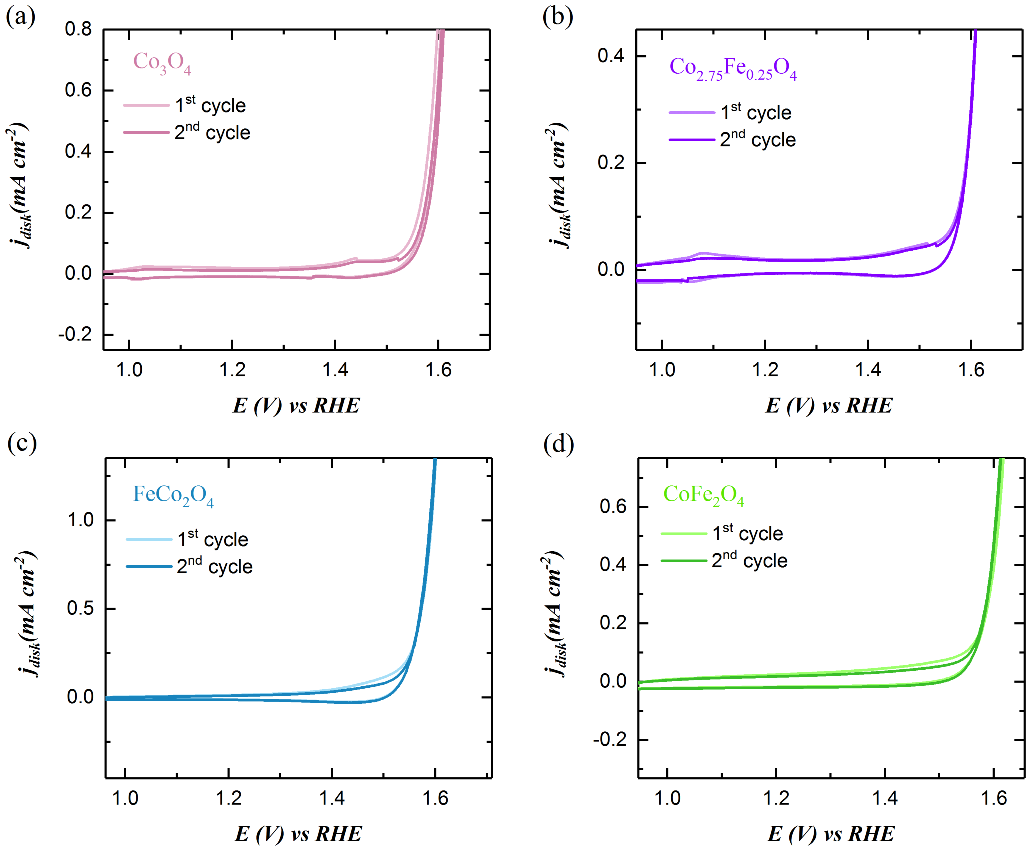

Supplementary Fig. 1. The 1st and 2nd CVs of pristine $Co_{3-x}Fe_xO_4$ spinel oxides in O_2-saturated 1 M KOH with a scan rate of 10 mV s^{-1}: (a) Co_3O_4; (b) $Co_{2.75}Fe_{0.25}O_4$; (c) $FeCo_2O_4$ and (d) $CoFe_2O_4$.

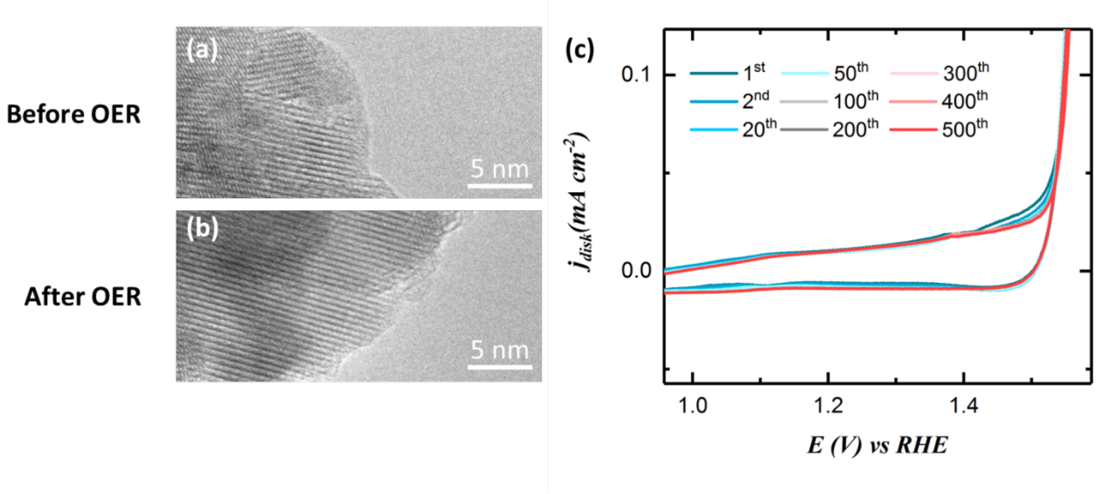

Supplementary Fig. 2. a, b, The HRTEM images of Pristine $CoFe_2O_4$ (a) before and (b) after 500 OER CVs in 1M KOH. **c,** The 500 CV cycles of $CoFe_2O_4$ of 10 mV s^{-1} in 1 M KOH. The OER CV shows negligible change during 500 cycles.

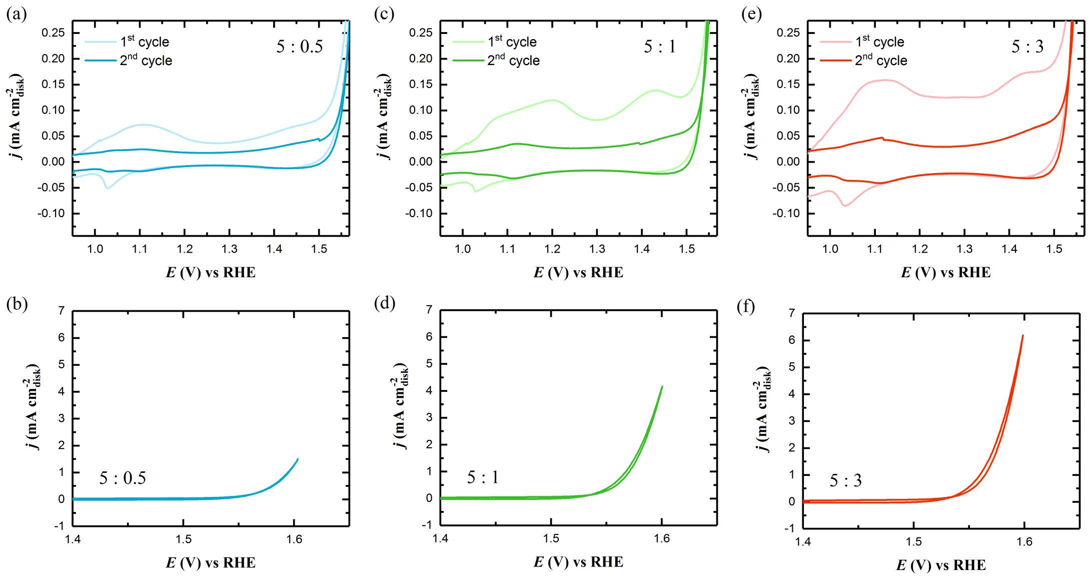

Supplementary Fig. 3. (a, c, e) The pseudo-capacitive region of the CV and (b, d, f) the 2nd cycle of OER CV of $Co_{2.75}Fe_{0.25}O_4$ (S) in different sulfurization degree: (a-b) 5:0.5, (c-d) 5:1 and (e-f) 5:3. The ratio denotes the mass ratio of oxide to sulfur powder in the mixture before the sulfurization.

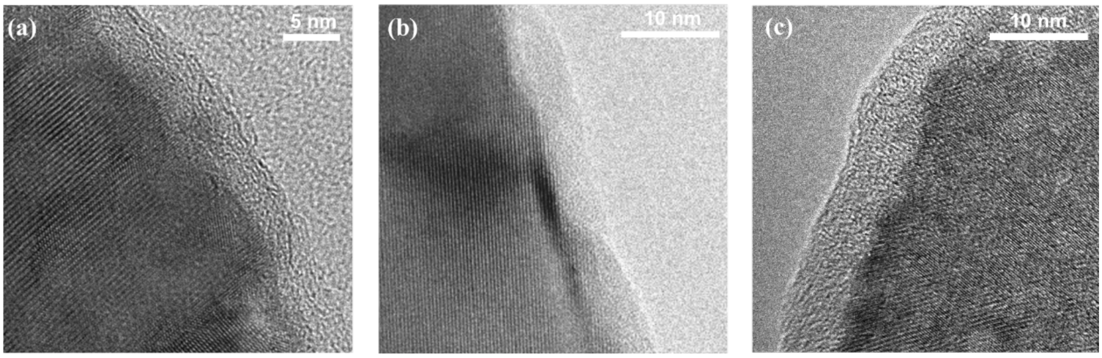

Supplementary Fig. 4. The HRTEM images of the $Co_{2.75}Fe_{0.25}O_4$/Co(Fe)O_xH_y reconstructed from $Co_{2.75}Fe_{0.25}O_4$ (S) with different sulfurization degree: (a) 5:0.5, (b) 5:1, and (c) 5:3. The ratio denotes the mass ratio of oxide to sulfur powder in the mixture before the sulfurization. No Nafion binder or carbon was added.

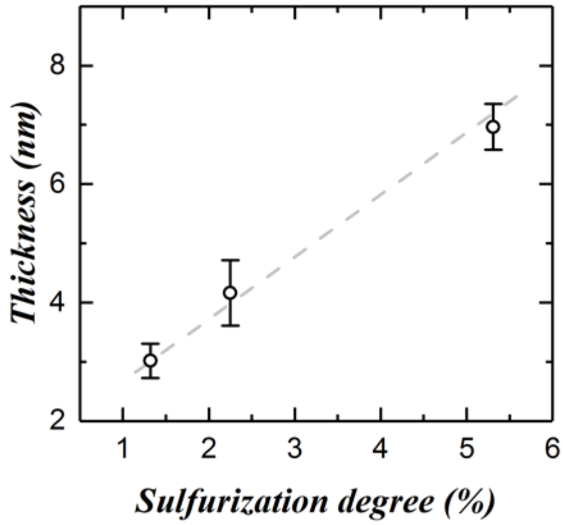

Supplementary Fig. 5. The plot of the thickness of oxyhydroxide layer after reconstruction versus the sulfidization degree of $Co_{2.75}Fe_{0.25}O_4$ oxide. The sulfidization degree of $Co_{2.75}Fe_{0.25}O_4$ oxide is determined by the ICP-OES measurement. The error bars represent the standard deviation from three independent measurements of the thickness of oxyhydroxide layer.

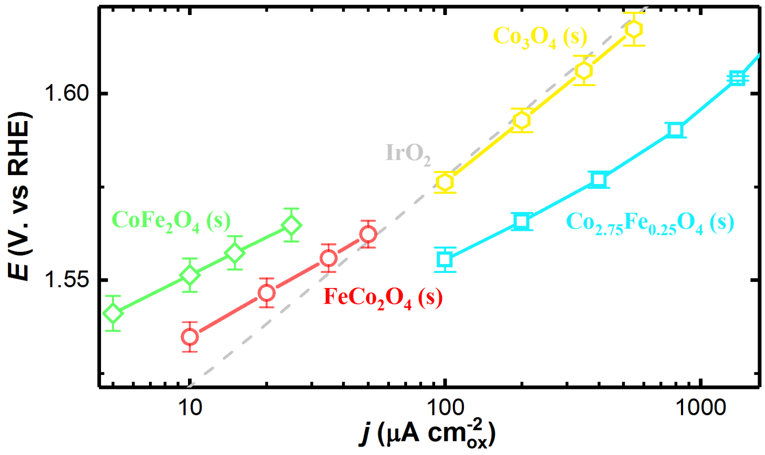

Supplementary Fig. 6. The Tafel plots in specific activity of $Co_{3-x}Fe_xO_4$ (s) (x=0~2.0), and IrO_2[6]. The plots are given after oxide surface area normalization, capacitance correction, and iR correction. The error bars represent the standard deviation from three independent measurements.

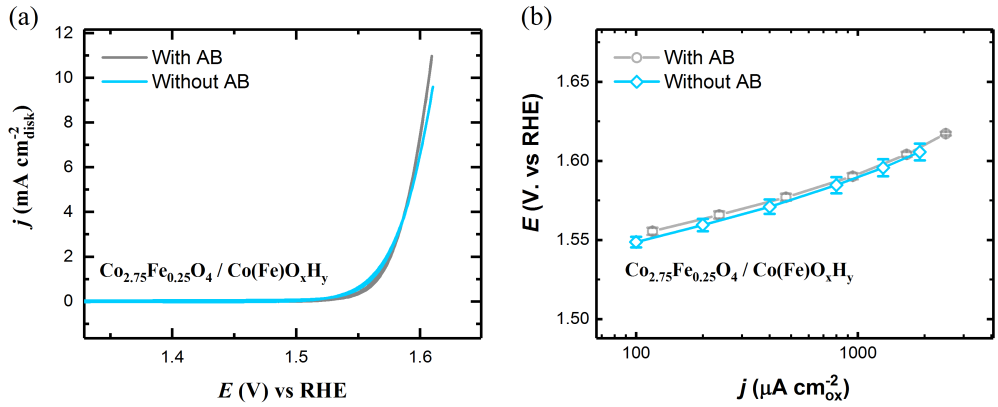

Supplementary Fig. 7. (a) The 2nd LSV of $Co_{2.75}Fe_{0.25}O_4$/Co(Fe)O_xH_y with and without mixing with carbon in O_2-saturated 1 M KOH. (b) The Tafel plots of the OER specific activity of $Co_{2.75}Fe_{0.25}O_4$/Co(Fe)O_xH_y with and without mixing with carbon. The error bar represents the standard deviation of three independent measurements.

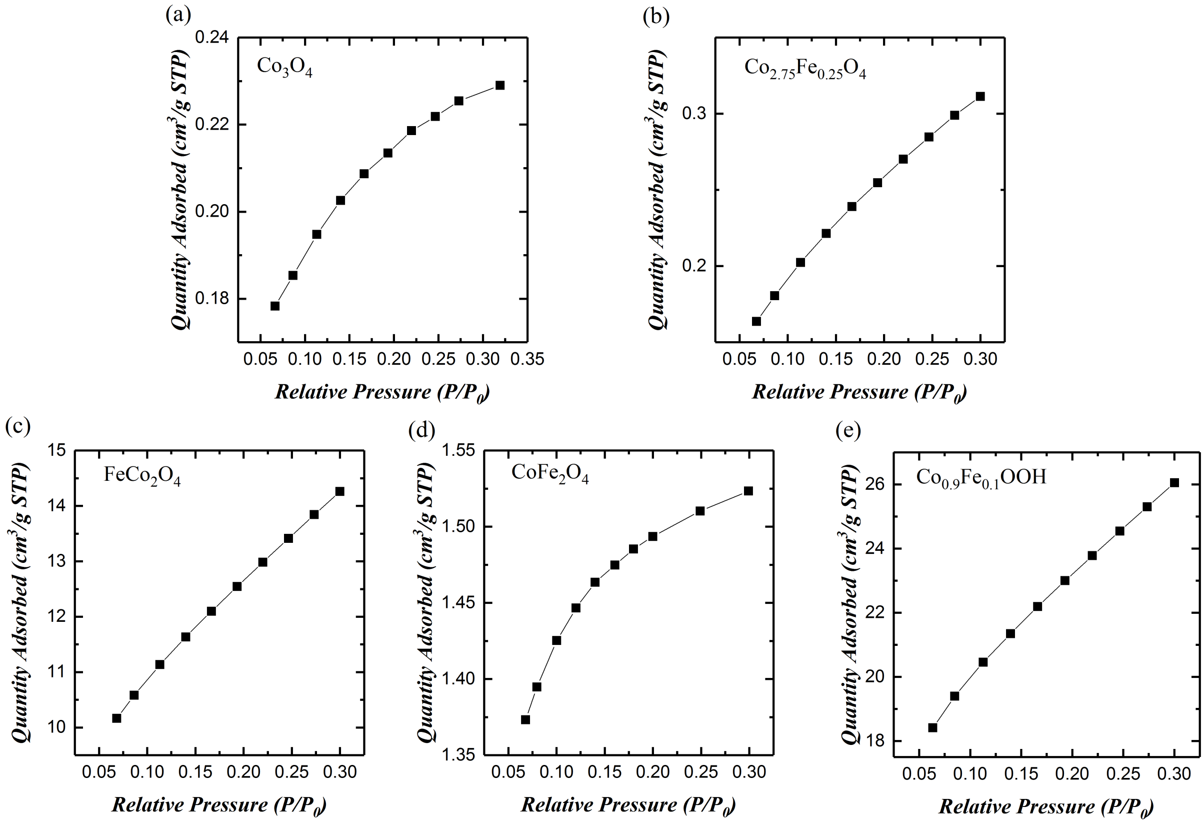

Supplementary Fig. 8. The BET surface area measurements of $Co_{3-x}Fe_xO_4$ spinel oxides. The details about surface area and materials synthesis parameters are summarized in Supplementary Table 1.

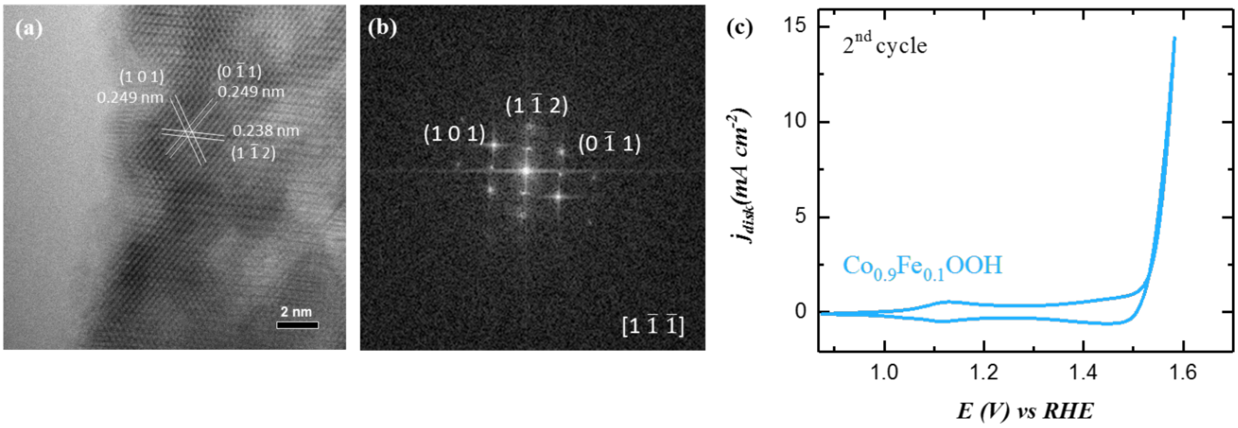

Supplementary Fig. 9. (a) The HRTEM image and (b) the fast Fourier transformed (FFT) pattern of as-prepared $Co_{0.9}Fe_{0.1}OOH$. (c) The cyclic voltammetry (CV) of $Co_{0.9}Fe_{0.1}OOH$ at the steady state with a scan rate of 10 mV s^{-1} in O_2-saturated 1 M KOH.

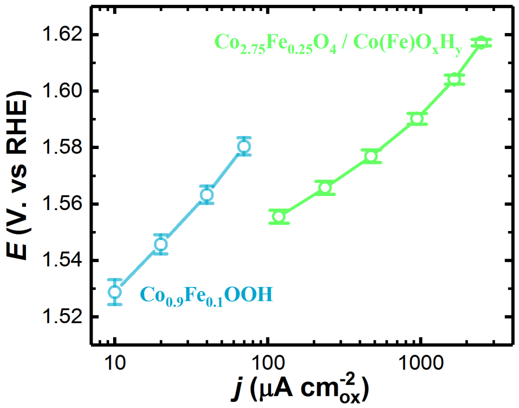

Supplementary Fig. 10. The Tafel plots of the OER specific activity of reconstructed $Co_{2.75}Fe_{0.25}O_4$(s) (i.e. $Co_{2.75}Fe_{0.25}O_4$ / $Co(Fe)O_xH_y$) and $Co_{0.9}Fe_{0.1}OOH$. The current density is normalized to the surface area of catalysts determined by BET measurement

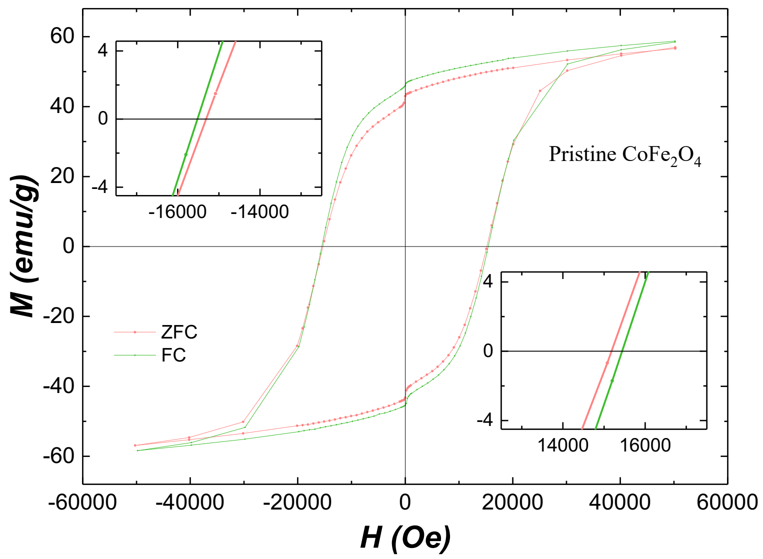

Supplementary Fig. 11. The magnetic hysteresis loop of pristine $CoFe_2O_4$ under both field-cooled (FC) mode and zero-field-cooled (ZFC) mode.

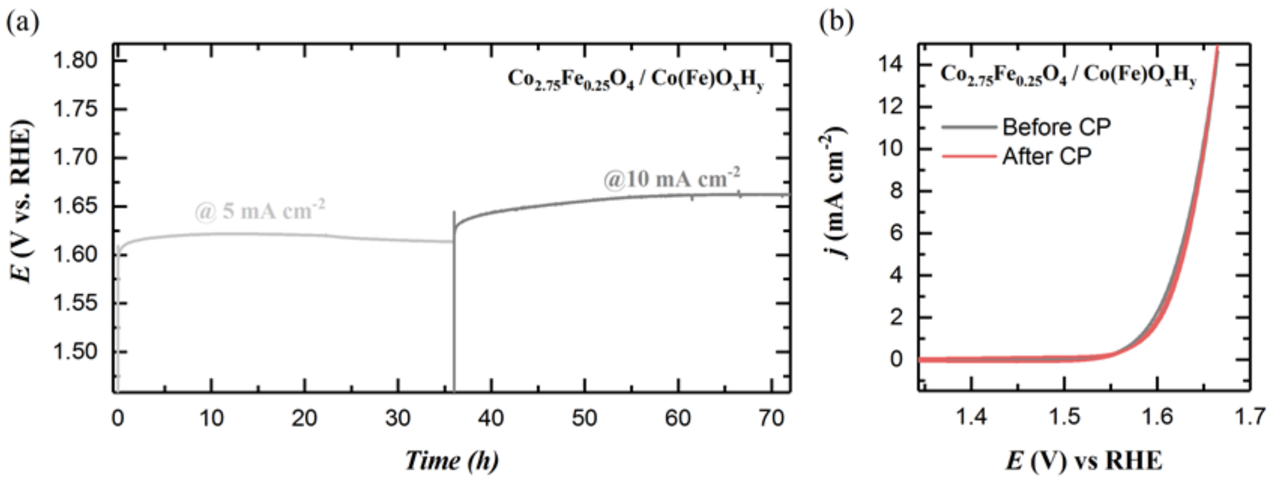

Supplementary Fig. 12. (a) The chronopotentiometry test of $Co_{2.75}Fe_{0.25}O_4/Co(Fe)O_xH_y$ for totally 72 hours under the OER current densities of 5.0 and 10.0 mA cm_{disk}^{-2} in 1 M KOH. (b) The CVs (scan rate of 10 mV s^{-1}) of $Co_{2.75}Fe_{0.25}O_4/Co(Fe)O_xH_y$) before and after CP measurement. The electrodes were fabricated without adding carbon.

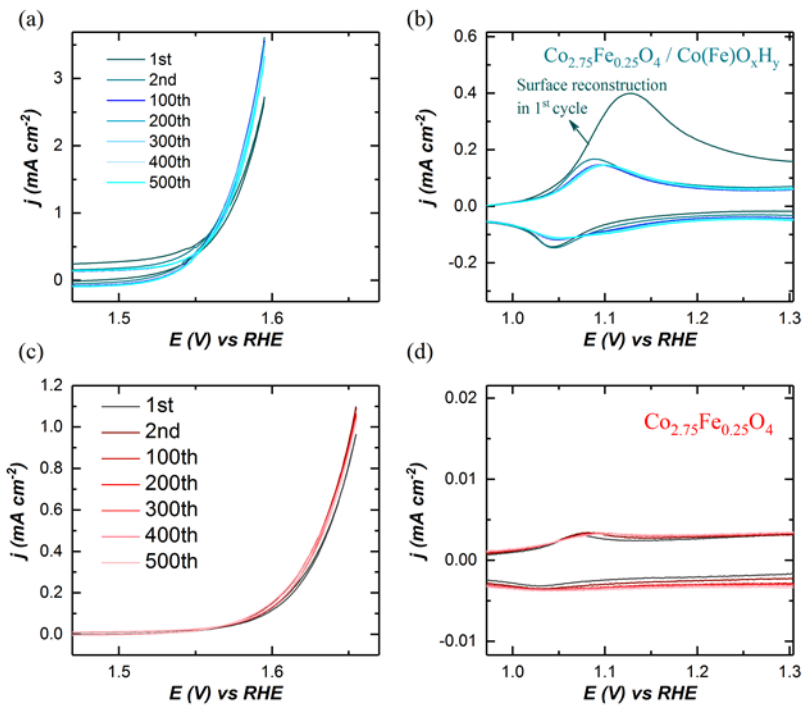

Supplementary Fig. 13. The 500 CVs of (a-b) $Co_{2.75}Fe_{0.25}O_4/Co(Fe)O_xH_y$ and (b-c) $Co_{2.75}Fe_{0.25}O_4$ at scan rate of 10 mV s^{-1} in 1 M KOH. In Figure (a) and (b), the 1st cycle involves the reconstruction from the pre-catalyst (sulfurized $Co_{2.75}Fe_{0.25}O_4$) to the desired catalyst ($Co_{2.75}Fe_{0.25}O_4/Co(Fe)O_xH_y$) and thus its CV profile is different from other subsequent cycles.

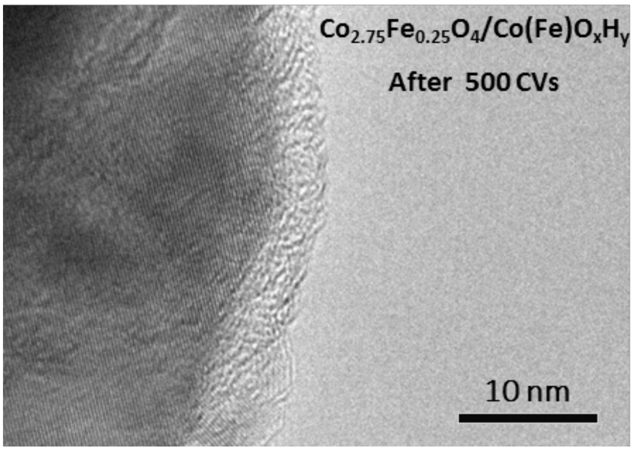

Supplementary Fig. 14. The HRTEM of Co2.75Fe0.25O4/Co(Fe)OxHy after 500 CV cycles.

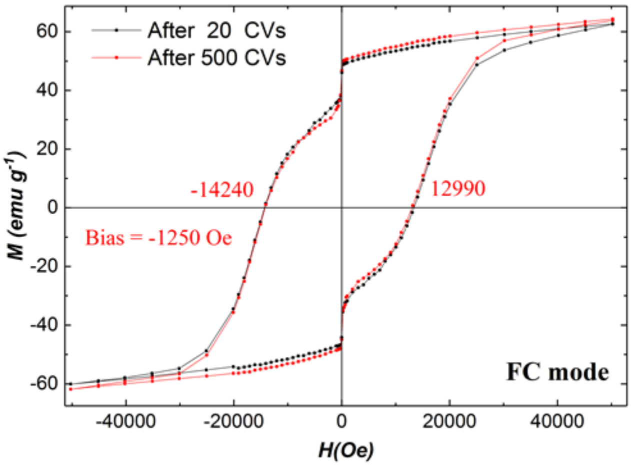

Supplementary Fig. 15. The hysteresis loop of $CoFe_2O_4/Co(Fe)O_xH_y$ under field-cooled (FC) mode after 20 and 500 CV cycles.

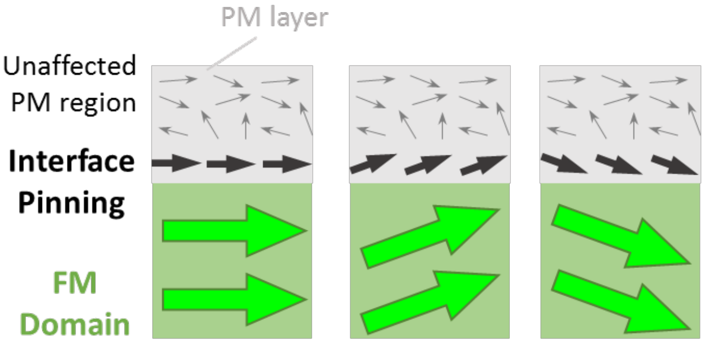

Supplementary Fig. 16. The schematic illustration of the spin pinning effect at the interface between ferromagnetic (FM) magnetic domains and the paramagnetic (PM) layer. The spins in a paramagnetic material are naturally disordered. The spins in the FM magnetic domains of a ferromagnetic materials are naturally highly aligned.

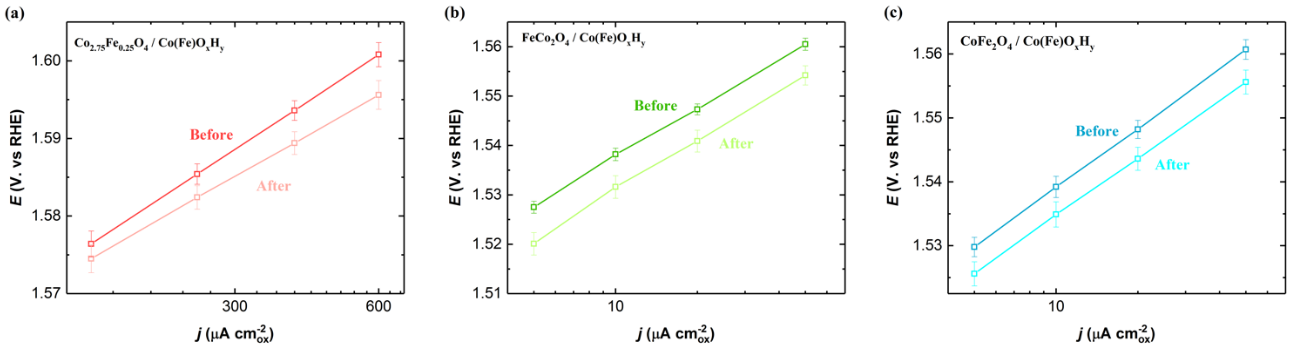

Supplementary Fig. 17. The Tafel plots of (a) $Co_{2.75}Fe_{0.25}O_4$/Co(Fe)O_xH_y, (b) $FeCo_2O_4$/Co(Fe)O_xH_y and (c) $CoFe_2O_4$/Co(Fe)O_xH_y before and after magnetization under 0.5 T for 15 min. The error bars represent the standard deviation from three independent measurements.

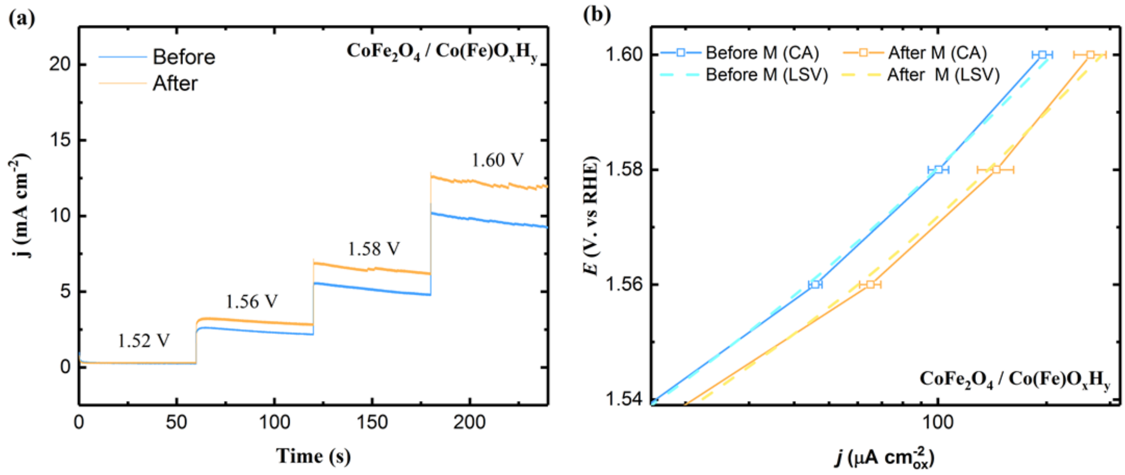

Supplementary Fig. 18. (a) The chronoamperometry of the reconstructed $CoFe_2O_4$ (i.e. $CoFe_2O_4$/Co(Fe)O_xH_y) before and after magnetization (0.5T, 15 min). (b) The OER Tafel plots of $CoFe_2O_4$/Co(Fe)O_xH_y determined by CA and LSV (scan rate of 10 mV s^{-1}) measurements. The error bars represent the standard deviation from three independent measurements

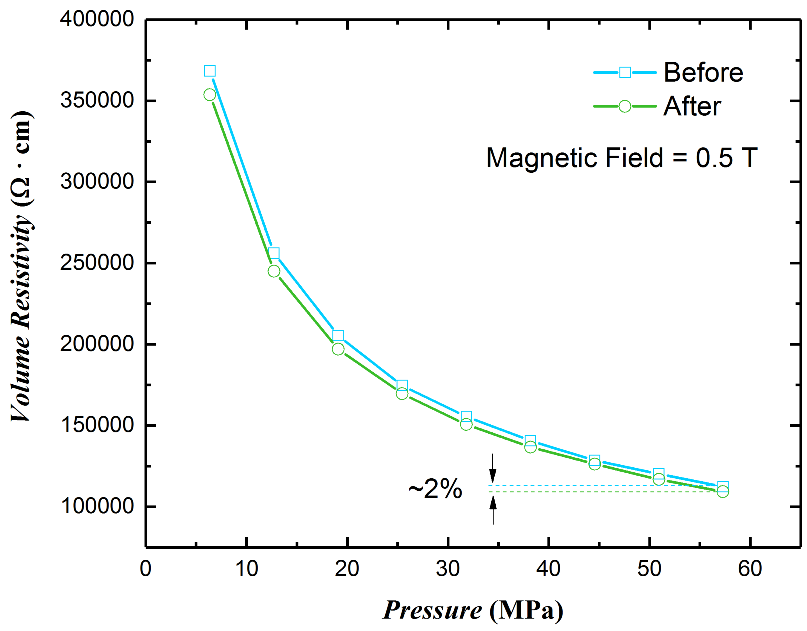

Supplementary Fig. 19. The magnetoresistance measurement of $CoFe_2O_4$ before and after applying magnetic field of 0.5 T for 15 min.

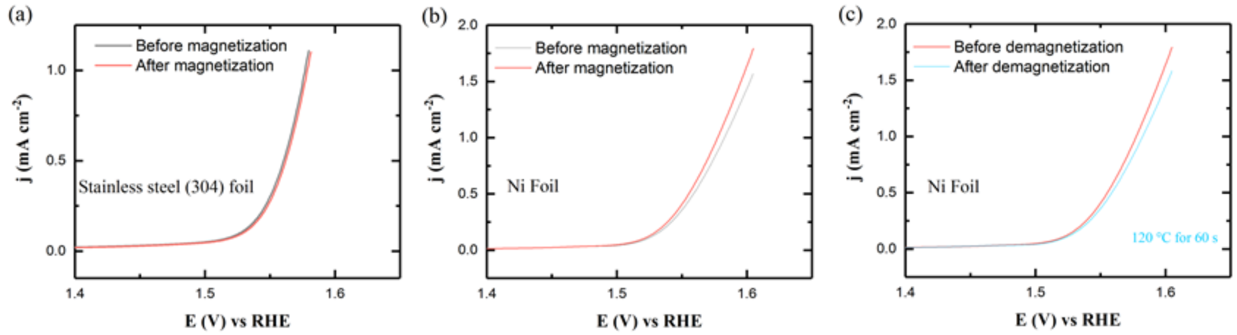

Supplementary Fig. 20. (a) The steady state (50th) LSV of stainless-steel (304) foil in 1 M KOH before magnetization and the LSV after magnetization (under 0.5 T for 15 min). (b) The steady state (50th) LSV of Ni foil in 1 M KOH and the LSV after magnetization (under 0.5 T for 15 min). (c) The LSV of magnetized Ni foil before and after demagnetization.

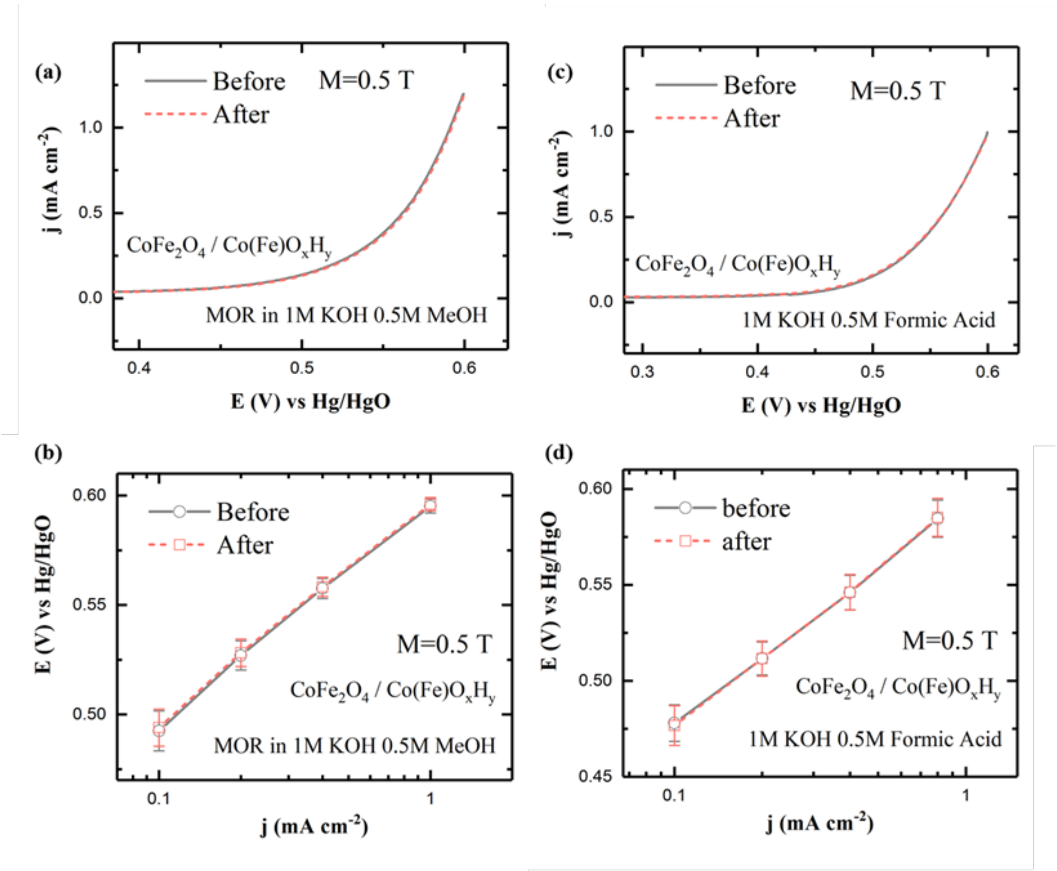

Supplementary Fig. 21. The electrochemical performance of $CoFe_2O_4$/Co(Fe)O_xH_y and the corresponding Tafel plots before and after magnetization under 0.5 T for 15 min for (a-b) methanol oxidation reaction (MOR) and (c-d) Formic acid oxidation (FOR). The error bars represent the standard deviation of at least three independent measurements.

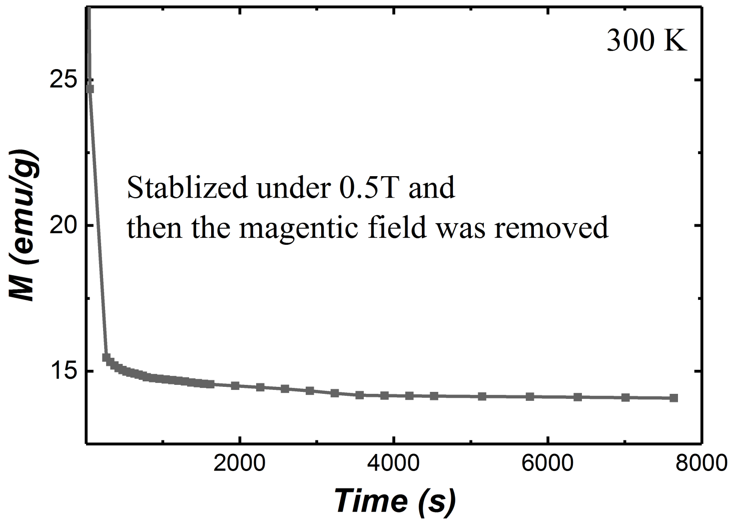

Supplementary Fig. 22. The magnetization of $CoFe_2O_4$/ Co(Fe)O_xH_y after removing a constant magnetic field of 0.5T.

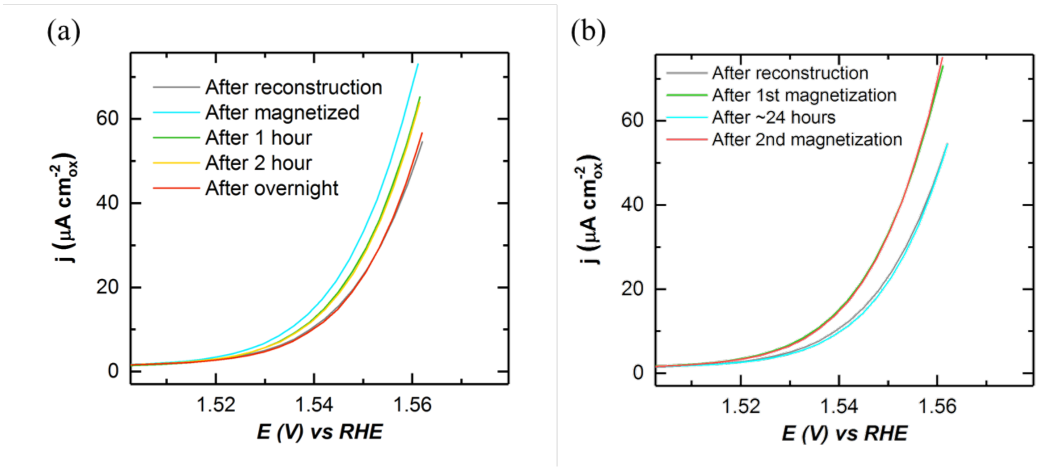

Supplementary Fig. 23. (a) The OER LSV of $CoFe_2O_4$ (s) after reconstruction, magnetization under 0.5T for 15 min, holding for 1 hour, holding for 2 hours and holding for overnight. (b) The re-activation of $CoFe_2O_4/Co(Fe)O_xH_y$ by magnetizing it again.

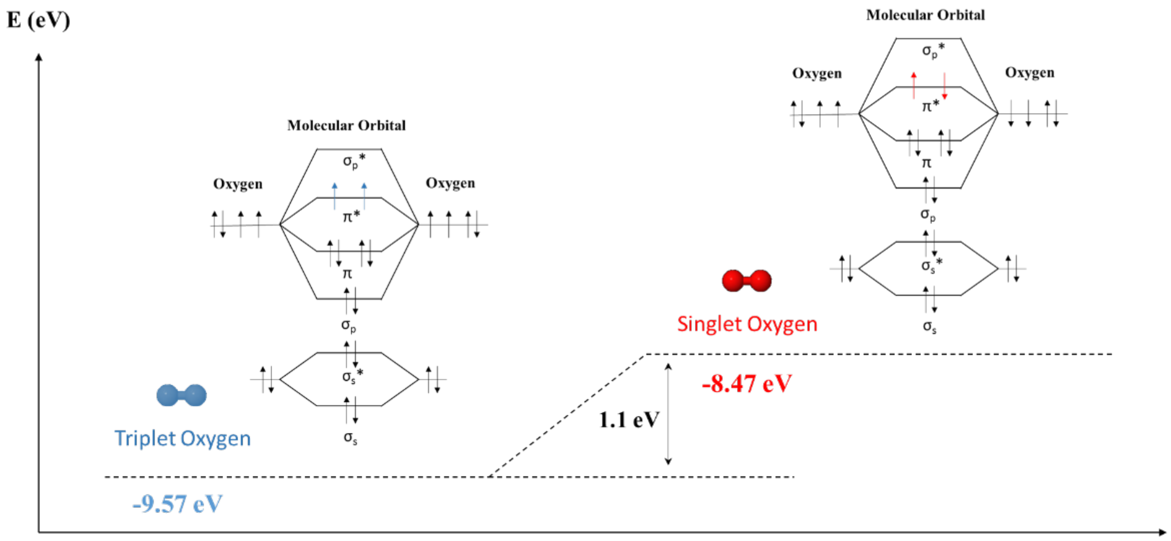

Supplementary Fig. 24. The energy diagram of generating triplet oxygen versus generating singlet oxygen.

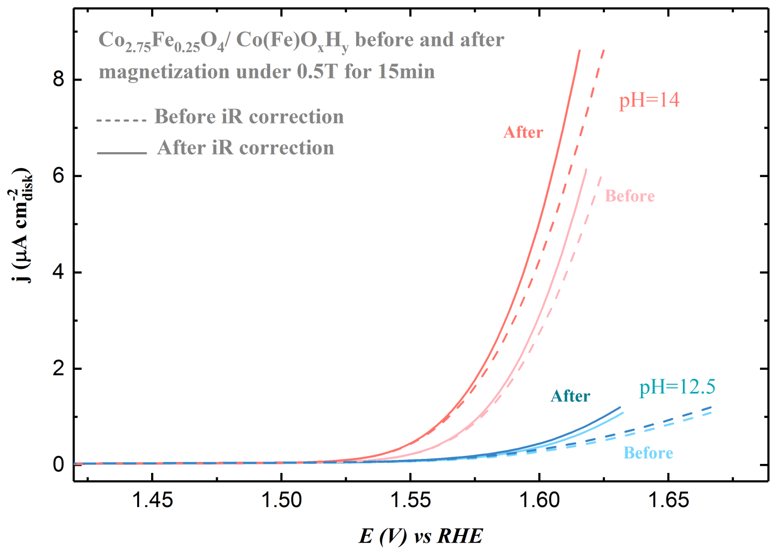

Supplementary Fig. 25. The iR correction of the OER LSV of $Co_{2.75}Fe_{0.25}O_4$ / $Co(Fe)O_xH_y$ in KOH electrolyte with different pH.

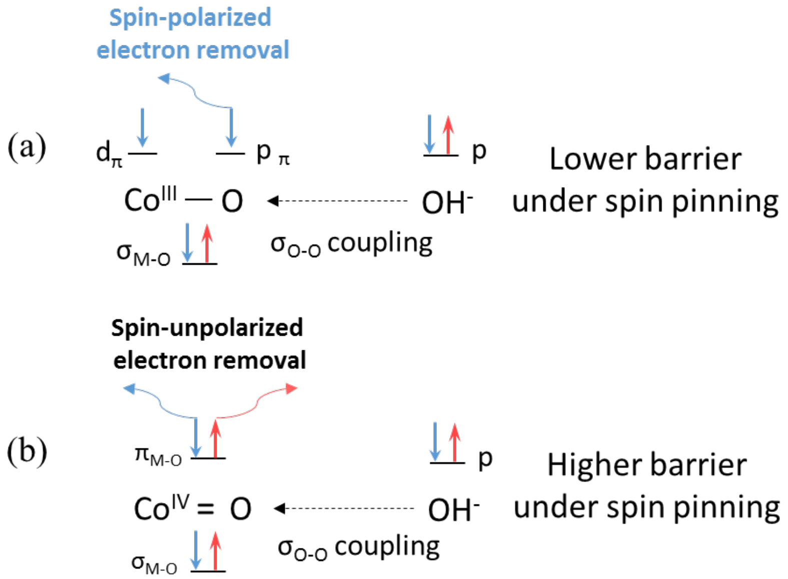

Supplementary Fig. 26. The alternative spin-related O-O coupling process under AEM (a) with Co-O· oxyl radical and (b) with Co=O oxo species

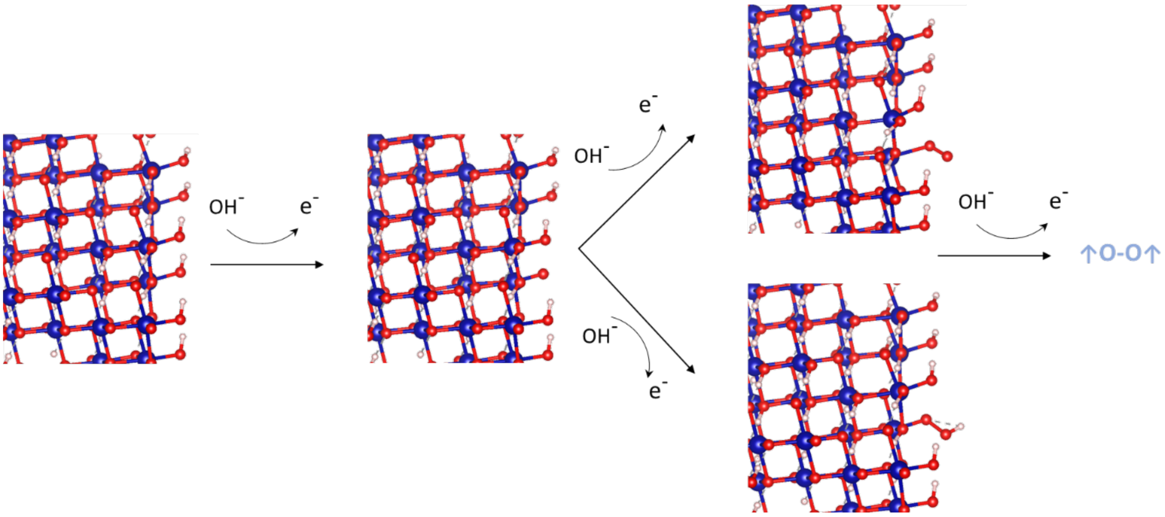

Supplementary Fig. 27. The slab models and coordinates for the DFT study of AEM and LOM paths.

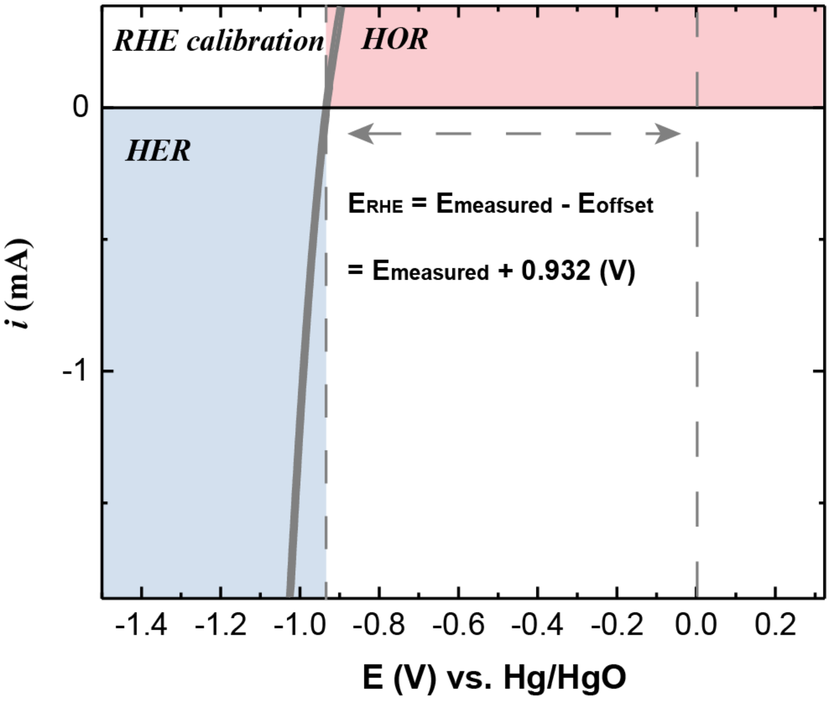

Supplementary Fig. 28. The RHE calibration of the Hg/HgO reference electrode in H_2-saturated 1 M KOH (pH=14.0).

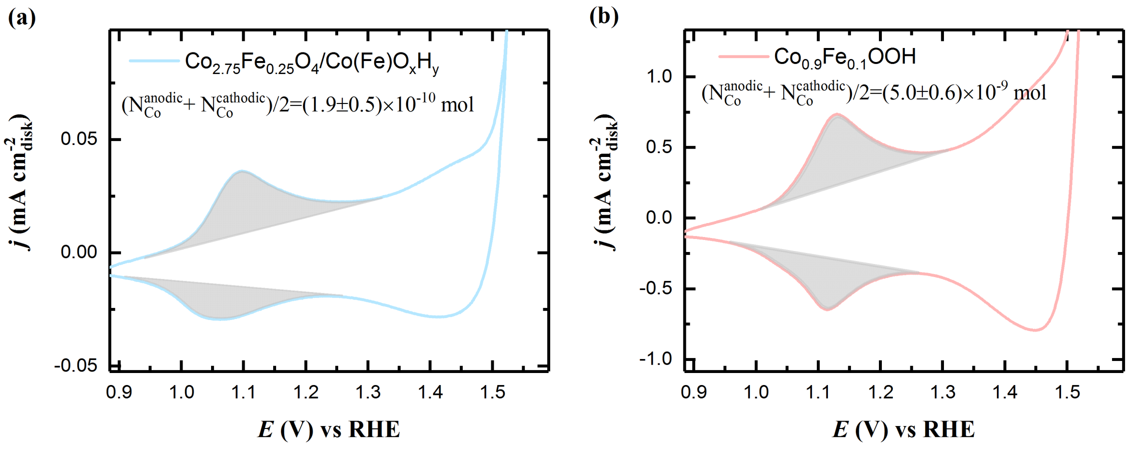

Supplementary Fig. 29. The Co^{2+}/Co^{3+} anodic and cathodic peaks in the CVs of (a) $Co_{2.75}Fe_{0.25}O_4/Co(Fe)O_xH_y$ and (b) $Co_{0.9}Fe_{0.1}OOH$. The N_{Co} is estimated by assuming a one-electron process.[7]

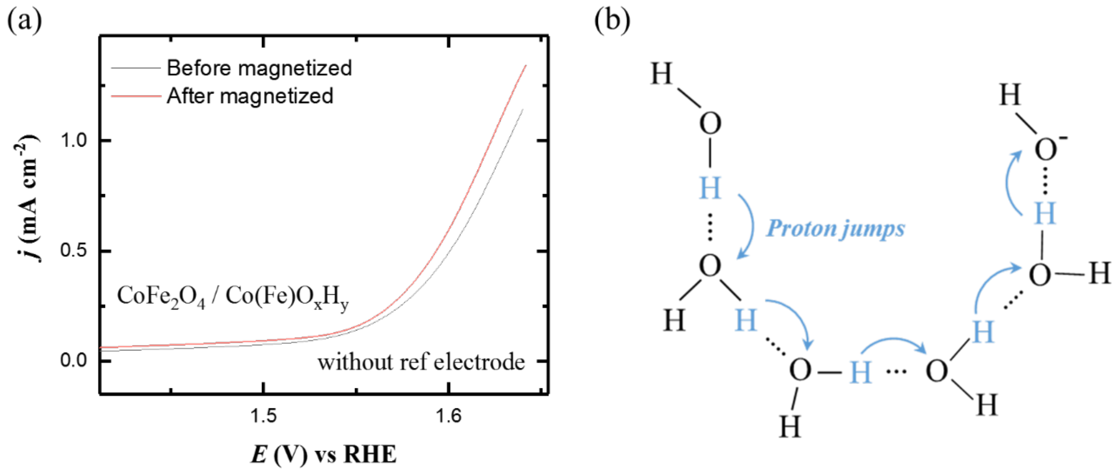

Supplementary Fig. 30. (a) The two-electrode measurement of $CoFe_2O_4/Co(Fe)O_xH_y$ before and after magnetization under 0.5 T for 15 min. The reference electrode is not used in the two-electrode system. (b) The mechanism of proton hopping (jump) for OH^- in aqueous solution.[8]

Supplementary Table 1. ICP result of $Co_{3-x}Fe_xO_4$ (s)

	Elemental ratio			Sulfurization degree
	Co%	Fe%	S%	
Co_3O_4 (s) (5:1)	97.09%	0	2.91%	2.06%
$Co_{2.75}Fe_{0.25}O_4$ (s) (5:0.5)	90.16%	8.11%	1.73%	1.32%
$Co_{2.75}Fe_{0.25}O_4$ (s) (5:1)	89.29%	8.04%	2.67%	2.25%
$Co_{2.75}Fe_{0.25}O_4$ (s) (5:3)	85.68%	7.71%	6.61%	5.31%
$FeCo_2O_4$ (s) (5:1)	64.52%	32.26%	3.23%	2.25%
$CoFe_2O_4$ (s) (5:1)	32.47%	63.96%	3.57%	2.75%

The ratio denotes the mass ratio of oxide to sulfur during the sulfurization

Supplementary Table 2. Summary synthesis parameters and surface areas (**A_s**) of $Co_{3-x}Fe_xO_4$ spinel oxides.

	**Calcination temperature (°C)**	**Calcination time**	**Mass for BET Measurement (g)**	**A_s (m^2 g^{-1})**	**STD**
Co_3O_4	600	6 hours	1.1799	0.81	0.02
$Co_{2.75}Fe_{0.25}O_4$	600	6 hours	0.705	1.10	0.08
$FeCo_2O_4$	400	6 hours	0.1717	43.16	1.23
$CoFe_2O_4$	500	6 hours	0.1436	19.17	0.51
$Co_{0.9}Fe_{0.1}OOH$	90	24 hours	0.3447	80.85	2.11

Supplementary Table 3. The correction of zero point energy and entropy of the adsorbed and gaseous species.

	ZPE(eV)	TS(eV)
*OOH	0.35	0
*O	0.05	0
*OH	0.31	0.01
H_2O	0.56	0.67
H_2	0.27	0.41

Supplementary Table 4. The magnetizations of Co and O in *OH, *O, *OOH and *OO

	***OH**	***O**	***OOH**	***OO**
O	0.343	0.828	0.373; 0.190	-0.725; -0.818
Co	2.958	1.126	0.332	2.579

Supplementary References

1. Burke, M. S., et al., Cobalt–Iron (Oxy)hydroxide Oxygen Evolution Electrocatalysts: The Role of Structure and Composition on Activity, Stability, and Mechanism. *J. Am. Chem. Soc.* **137**, 3638-3648 (2015).
2. Li, Y., et al., One-Step Synthesis of a Coral-Like Cobalt Iron Oxyhydroxide Porous Nanoarray: An Efficient Catalyst for Oxygen Evolution Reactions. *ChemPlusChem* **84**, 1681-1687 (2019).
3. Lee, D. H., et al., Asymmetric Transport Mechanisms of Hydronium and Hydroxide Ions in Amorphous Solid Water: Hydroxide Goes Brownian while Hydronium Hops. *The Journal of Physical Chemistry Letters* **5**, 2568-2572 (2014).
4. Lin, M.-Y.; Hourng, L.-W.; Kuo, C.-W., The effect of magnetic force on hydrogen production efficiency in water electrolysis. *International Journal of Hydrogen Energy* **37**, 1311-1320 (2012).
5. Matsushima, H.; Iida, T.; Fukunaka, Y., Gas bubble evolution on transparent electrode during water electrolysis in a magnetic field. *Electrochimica Acta* **100**, 261-264 (2013).
6. Lee, Y., et al., Synthesis and Activities of Rutile IrO2 and RuO2 Nanoparticles for Oxygen Evolution in Acid and Alkaline Solutions. *J. Phys. Chem. Lett.* **3**, 399-404 (2012).
7. Surendranath, Y.; Kanan, M. W.; Nocera, D. G., Mechanistic Studies of the Oxygen Evolution Reaction by a Cobalt-Phosphate Catalyst at Neutral pH. *Journal of the American Chemical Society* **132**, 16501-16509 (2010).
8. Miyake, T. & Rolandi, M. Grotthuss mechanisms: from proton transport in proton wires to bioprotonic devices. *J Phys Condens Matter* **28**, 023001 (2016).
